# DUF2285 is a novel helix-turn-helix domain variant that orchestrates both activation and antiactivation of conjugative element transfer in proteobacteria

**DOI:** 10.1093/nar/gkad457

**Published:** 2023-05-29

**Authors:** William J Jowsey, Calum R P Morris, Drew A Hall, John T Sullivan, Robert D Fagerlund, Karina Y Eto, Paul D Solomon, Joel P Mackay, Charles S Bond, Joshua P Ramsay, Clive W Ronson

**Affiliations:** Department of Microbiology and Immunology, University of Otago, Dunedin 9016, New Zealand; Department of Microbiology and Immunology, University of Otago, Dunedin 9016, New Zealand; School of Molecular Sciences, University of Western Australia, 35 Stirling Highway, Crawley, WA 6009, Australia; Curtin Medical School and Curtin Health Innovation Research Institute, Curtin University, Perth, WA 6102, Australia; Department of Microbiology and Immunology, University of Otago, Dunedin 9016, New Zealand; Department of Microbiology and Immunology, University of Otago, Dunedin 9016, New Zealand; Curtin Medical School and Curtin Health Innovation Research Institute, Curtin University, Perth, WA 6102, Australia; School of Life and Environmental Sciences, University of Sydney, Sydney, NSW 2006, Australia; School of Life and Environmental Sciences, University of Sydney, Sydney, NSW 2006, Australia; School of Molecular Sciences, University of Western Australia, 35 Stirling Highway, Crawley, WA 6009, Australia; Marshall Centre for Infectious Disease Research and Training, The University of Western Australia, 35 Stirling Highway, Crawley, WA 6009, Australia; Curtin Medical School and Curtin Health Innovation Research Institute, Curtin University, Perth, WA 6102, Australia; Department of Microbiology and Immunology, University of Otago, Dunedin 9016, New Zealand

## Abstract

Horizontal gene transfer is tightly regulated in bacteria. Often only a fraction of cells become donors even when regulation of horizontal transfer is coordinated at the cell population level by quorum sensing. Here, we reveal the widespread ‘domain of unknown function’ DUF2285 represents an ‘extended-turn’ variant of the helix-turn-helix domain that participates in both transcriptional activation and antiactivation to initiate or inhibit horizontal gene transfer. Transfer of the integrative and conjugative element ICE*Ml*Sym^R7A^ is controlled by the DUF2285-containing transcriptional activator FseA. One side of the DUF2285 domain of FseA has a positively charged surface which is required for DNA binding, while the opposite side makes critical interdomain contacts with the N-terminal FseA DUF6499 domain. The QseM protein is an antiactivator of FseA and is composed of a DUF2285 domain with a negative surface charge. While QseM lacks the DUF6499 domain, it can bind the FseA DUF6499 domain and prevent transcriptional activation by FseA. DUF2285-domain proteins are encoded on mobile elements throughout the proteobacteria, suggesting regulation of gene transfer by DUF2285 domains is a widespread phenomenon. These findings provide a striking example of how antagonistic domain paralogues have evolved to provide robust molecular control over the initiation of horizontal gene transfer.

## Graphical Abstract

**Graphical Abstract:**
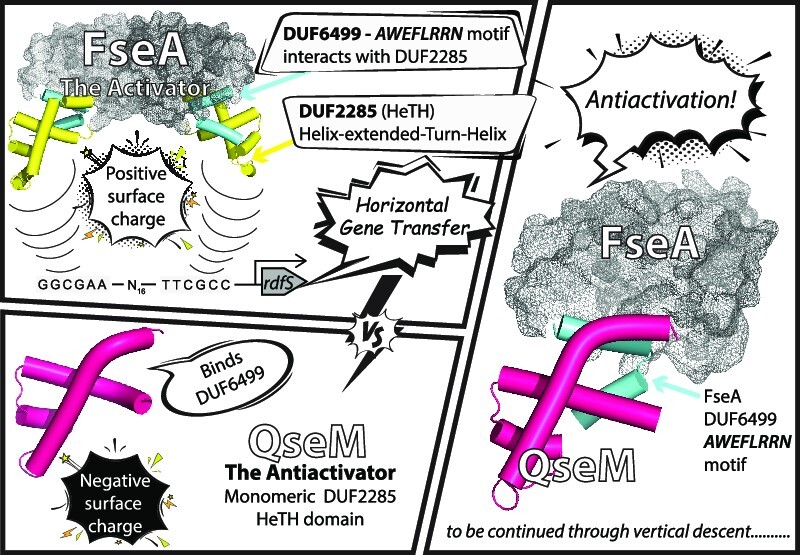

## INTRODUCTION

Mobile genetic elements drive bacterial evolution through the horizontal gene transfer of adaptive traits that allow their hosts to colonize specific niches (1). Integrative and conjugative elements (ICEs) are a common class of mobile genetic element that are maintained in the host chromosome. When induced for transfer, ICEs excise as circular DNA elements before transfer by conjugation followed by chromosomal reintegration in both host and recipient. Traits encoded by well-characterized ICEs include antibiotic resistance, catabolism of xenobiotic compounds, and determinants of pathogenesis and symbiosis (2,3).

The symbiosis island of the soil bacterium *Mesorhizobium japonicum* R7A (ICE*Ml*Sym^R7A^) is a 502-kb ICE that transfers to non-symbiotic *Mesorhizobium* sp. strains, rendering them capable of forming a nitrogen-fixing symbiosis with legume hosts (4,5). ICE*Ml*Sym^R7A^ integrates into the 3′-end of the chromosomal *phe-*tRNA gene through the action of the site-specific recombinase IntS. Subsequent transfer requires the expression of the recombination directionality factor RdfS, which stimulates IntS to catalyse excision of ICE*Ml*Sym^R7A^ (6,7). Excision and transfer of ICE*Ml*Sym^R7A^ is stimulated by the quorum-sensing (QS) regulator TraR, which in the presence of *N*-acyl homoserine lactone signalling molecules activates expression of the ICE*Ml*Sym^R7A^ transcriptional activator FseA (7,8). FseA then activates expression of RdfS to initiate excision of ICE*Ml*Sym^R7A^ (9).

The FseA protein is encoded by two overlapping open reading frames, *msi172* and *msi171*. During translation of the *msi172-msi171* mRNA, a low-frequency + 1 programmed ribosomal frameshift (PRF) fuses the *msi172* and *msi171* coding sequences to produce the FseA polypeptide (9). FseA contains two domains of unknown function (DUF), the N-terminal DUF6499 and the C-terminal DUF2285. Genes encoding FseA-like proteins and the conserved + 1 PRF site are found throughout the γ, β and α-proteobacteria, but are frequently misannotated or unannotated (7). While FseA shares only weak primary sequence similarities with known DNA-binding proteins, genetic experiments indicate FseA is likely a direct activator of the *rdfS* promoter (P*rdfS*) that may bind a conserved inverted repeat (IR) sequence present immediately upstream of the P*rdfS* -35 element (9).

Although ICE*Ml*Sym^R7A^ excision and horizontal transfer can be activated by QS and FseA, only a minority of cells in R7A populations respond to N-acyl homoserine lactone and participate as donors of ICE*Ml*Sym^R7A^. In R7A populations most cells are inhibited for QS and ICE*Ml*Sym^R7A^ excision and transfer by the antiactivator protein QseM (8). The remaining cells are repressed for *qseM* transcription by a bistable epigenetic switch, which allows for a small proportion of the population to participate in QS and initiate ICE*Ml*Sym^R7A^ excision and transfer (10). QseM contains a lone DUF2285 domain that shares ∼18% amino acid identity with the FseA DUF2285 domain. Bacterial two-hybrid experiments show QseM directly interacts with the *msi172*-encoded portion of FseA (composed of DUF6499) and, independently, with TraR in the presence of *N*-acyl homoserine lactone (8,9). In summary, QseM through its dual target antiactivation of TraR and FseA is the critical factor determining the ability of cells to become epigenetically activated for QS and ICE*Ml*Sym^R7A^ transfer.

Here, we show that purified MBP-tagged FseA forms a homodimer in solution and binds to DNA containing the IR region upstream of P*rdfS*. The entire IR and the inverted orientation of its repeats are critical for FseA-dependent transcriptional activation. Computational prediction of the FseA structure suggests that the DUF2285 domain folds into a distinct variant of the DNA-binding helix-turn-helix (HTH) domain that deviates from the canonical HTH domain by containing an ‘extended turn’ motif. The FseA DUF2285 domain is also predicted to make core interdomain contacts with α-helix two of the FseA DUF6499 domain. Conserved residues in both DUF domains are critical for activation of P*rdfS*, and residues that make up a positively charged surface of the DUF2285 domain are critical for DNA binding. We determined the structure of QseM by nuclear magnetic resonance (NMR), revealing that monomeric QseM also contains an extended-turn variant of the HTH domain akin to the FseA DUF2285 domain prediction. QseM binds α-helix two of the FseA DUF6499 domain and likely mimics the key contacts formed between the FseA DUF2285 domain and DUF6499 domain. QseM has an overall negatively charged surface and is unable to bind P*rdfS* DNA. Therefore, QseM appears to have evolved to become an antiactivator of FseA that has lost DNA-binding ability but retained the ability to bind the DUF6499 domain α-helix two of FseA.

## MATERIALS AND METHODS

### Strains, plasmids, and growth media

*Mesorhizobium japonicum* and *Escherichia coli* strains used in this study are listed in Supplementary Table S1. Plasmids used in this study are listed in Supplementary Table S2. Bacterial strains were cultured as previously described (6,7,11). Where appropriate, media were supplemented with antibiotics at the following concentrations: ampicillin (Ap) 100 μg/ml, chloramphenicol (Cm) 50 μg/ml, kanamycin (Km) 50 μg/ml, gentamicin (Gm) 50 μg/ml (*E. coli*) and 25 μg/ml (*M. japonicum*), tetracycline (Tc) 10 or 15 μg/ml (*E. coli*) and 2 μg/ml (*M. japonicum*). Medium used to grow *E. coli* ST18 was supplemented with 50 μg/ml of 5-aminolevulinic acid.

### Cloning

DNA cloning was carried out using Gibson assembly (New England BioLabs) according to the manufacturer's instructions. Gene mutations or truncations were generated with synthesized DNA oligonucleotides or using PCR. PCR-based mutagenesis was carried out with DNA primers incorporating mis-matched base pairs compared to the wild-type gene template DNA. For truncations, DNA primers bound to template sequence sites that excluded either upstream or downstream sequence in the amplified product. PCR amplification of DNA for cloning was carried out using Phusion DNA polymerase (New England BioLabs) according to the manufacturer's instructions. All constructs were confirmed using Sanger sequencing (Massey University Genome Service). Conjugation of plasmids from *E*.*coli* ST18 to *M. japonicum* R7ANS was performed by biparental spot mating as previously described (12).

### β-Galactosidase assays

Broths inoculated from single colonies of *M. japonicum* R7ANS cells (R7A cured of ICE*Ml*Sym^R7A^, thereby avoiding possible interference from ICE genes) carrying pSDZ-P*rdfS*-*lacZ* or derivatives, or pSDZ-P*rdfS-lacZ* and pPR3G or its derivatives were grown for ∼72 h. One hundred microliters of culture was inoculated into fresh medium with or without 1 mM isopropyl β-d-1-thiogalactopyranoside (IPTG) and grown for 18–20 h. Cell density was estimated by absorbance at OD_600_, and cells were analysed for β-galactosidase expression using ortho-nitrophenyl-β-galactoside as previously described (13).

### Protein expression and purification

6H-MBP-FseA and 6H-MBP-FseA_R247A-R248A_ were expressed from pETM-41 in *E. coli* strain NiCo31(DE3). An overnight LB culture containing Km was used to inoculate 500 ml of LB containing Km, and the culture grown at 37°C to an OD_600_ of ∼0.3. The temperature was reduced to 18°C and the culture further grown to an OD_600_ of 0.6, at which point IPTG was added to a final concentration of 1 mM. After shaking overnight at 180 rpm, cells were harvested by centrifugation. Cell pellets were resuspended in binding buffer (50 mM Na_2_HPO_4_/NaH_2_PO_4_ (combined to final pH of 6.35), 10% (v/v) glycerol, 500 mM NaCl, 20 mM imidazole), and supplemented with one cOmplete EDTA-free Protease Inhibitor Cocktail tablet (Roche) and 20 μg/ml DNaseI before lysis by five cycles through a French Press (Homogenising Systems) at 10 000 psi. Soluble lysate was separated by centrifugation at 4°C for 30–45 min at 15 000 × g and then loaded onto a 1 ml HisTrap FF column (GE Healthcare) pre-equilibrated with binding buffer using a ÄKTA pure chromatography system (GE Healthcare). Recombinant protein was eluted using a linear imidazole gradient to 100% elution buffer (50 mM Na_2_HPO_4_/NaH_2_PO_4_ (combined to final pH of 6.35 or 7.5 for 6H-MBP-FseA and 6H-MBP-FseA_R247A-R248A_ respectively), 10% (v/v) glycerol, 500 mM NaCl, 500 mM imidazole). Purified recombinant protein was pooled and centrifuged at 20 000 × g for 5 min at 4°C before further purification by size exclusion chromatography (SEC) using a HiLoad 16/600 Superdex 200 column (GE Healthcare) pre-equilibrated with SEC buffer (50 mM Na_2_HPO_4_/NaH_2_PO_4_ (combined to final pH of 6.35), 10% (v/v) glycerol, 500 mM NaCl). Fractions containing purified protein were pooled and stored at –80°C in 20–50 μl aliquots until use. 6H-MBP-FseA was stored at 0.73 μM final concentration. 6H-MBP-FseA_R247A-R248A_ was concentrated to 30 μM using a Vivaspin 6 MWCO 10 000 (Cytiva) column pre-equilibrated with SEC buffer before storage.

For use in electrophoretic mobility shift assays (EMSA), 6H-QseM was expressed and purified following the method above except that pETM-11 was used as the host vector, all buffers were at pH 7.5, and a Superdex 75 Increase 10/300 GL column (GE Healthcare) was used for SEC.

For use in NMR experiments, 6H-QseM was expressed from pQE80 in *E. coli* strain BL21(DE3)pLysS. A 5-ml overnight culture was grown at 37°C with Ap. The culture was used to inoculate 1 l of M9 minimal medium containing Ap, which was grown at 37°C with shaking for 12–24 h with induction by 0.2 mM IPTG. M9 minimal medium with 0.02 M ^13^C-glucose and 9.3 mM ^15^NH_4_Cl was used to express 6H-QseM with ^13^C and ^15^N necessary for many of the multidimensional NMR acquisitions. Cells were harvested by centrifugation at 4°C for 20 min at 10 000 × g and resuspended in NMR binding buffer (100 mM Na_2_HPO_4_/NaH_2_PO_4_ (combined to final pH of 7.5), 300 mM NaCl, 100 mM imidazole, 5–10% (v/v) glycerol). Cells were lysed using a Cell Disruptor CF (Constant Systems, UK) at 20 000 psi. The lysate was centrifuged at 4°C for 45 min at 15 000 × g, then passed through a 0.2 μm filter. Filtered lysate was loaded onto a 5-ml HisTrap HP column (GE Healthcare) pre-equilibrated with NMR binding buffer using a peristaltic pump (Bio-Rad) at a flow rate of 1–2 ml/min. 6H-QseM was purified using a ÄKTA pure chromatography system, and a linear imidazole gradient to 100% elution buffer (100 mM Na_2_HPO_4_/NaH_2_PO_4_ (combined to final pH of 7.5), 300 mM NaCl, 800 mM imidazole, 5–10% (v/v) glycerol). SEC was performed with a Superdex 200 16/600 column (GE Healthcare) pre-equilibrated with NMR SEC buffer (10 mM NaH_2_PO_4_, 20 mM NaCl (pH 7.5)). Protein was concentrated to 1–2 mg/ml using centrifugal filtration tubes (GE Healthcare, Millipore) prior to storage at –80°C in 200–300 μl aliquots.

### Electrophoretic mobility shift assays

PCR amplification of DNA for EMSAs was carried out using Phusion DNA polymerase (New England BioLabs) and the primers listed in Supplementary Table S3. For the synthesis of fluorescent P*rdfS* DNA, 5′-IRDye700-tagged primers and a template of 1 ng/μl of a pure 510-bp DNA fragment amplified from pSDZ-P*rdfS* were used in the PCR program: 98°C for 30 s; 35 cycles of 98°C for 10 s, 68°C for 15 s, then 72°C for 10 s; 72°C for 5 min. Glycerol was added to the product at 15–20% (v/v), followed by purification by TAE agarose (3% (w/v)) gel electrophoresis (2 h at 65 V).

EMSA reactions with 6H-MBP-FseA alone were carried out in 10 μl volumes containing 10 mM Na_2_HPO_4_/NaH_2_PO_4_ (combined to final pH of 6.35), 220 mM NaCl, 6% (v/v) glycerol, 1 mM DTT, 0.01 μg/μl poly(dI.dC), 0.1 μg/μl herring sperm DNA, 5 nM fluorescent DNA probe, 0.1–0.19 μg/μl BSA, and denoted purified protein concentrations. Where appropriate, excess unlabelled DNA was added to a final concentration of 260 nM and pre-incubated with protein for 30 min at 28°C prior to adding the fluorescent P*rdfS* DNA. Binding reactions were incubated at 28°C for 30 min. Samples were loaded onto a 4% (v/v) polyacrylamide gel (19:1 acrylamide/bis solution (Bio-Rad), 0.01% (v/v) TEMED, 0.02% (v/v) of 10% ammonium persulfate, 0.5 × TBE (45 mM Tris, 45 mM boric acid, and 1.25 mM EDTA (pH 8.3)) that was pre-run for at least 30 min. Gel electrophoresis was performed at 100 V for 50 min and fluorescent DNA imaged at 700 nm using an Odyssey Fc imaging system (LI-COR Biosciences) with Image Studio (version 5.2) (LI-COR Biosciences). Image Studio Lite (version 5.2) was used to quantitate protein-bound fluorescent DNA. The *K*_D_ was determined with the ratio of bound to unbound DNA from three independent replicates using the non-linear regression analysis specific binding with Hill slope in GraphPad Prism (version 9.1.2). Co-purified fluorescent P*rdfS* DNA that remained equally unbound at each 6H-MBP-FseA concentration was excluded from the analysis.

For EMSAs containing both QseM and FseA, assays were performed as stated above with the buffer conditions: 19 mM Na_2_HPO_4_/NaH_2_PO_4_, 190 mM NaCl, 9.8% (v/v) glycerol, 2 mM DTT, 0.02 μg/μl poly(dI.dC), 5 nM fluorescent DNA probe, 0.05–0.109 μg/μl BSA, and 6H-MBP-FseA concentrations of 26 or 436 nM and 6H-QseM concentrations of 5–4981 nM. QseM was incubated with the P*rdfS* DNA for 30 min before 6H-MBP-FseA was added.

### Compilation of the FseA homologue database

FseA homologue sequences were acquired using PSI-BLAST where searches were performed with FseA against non-redundant protein translations (GenBank CDS translations + PDB + SwissProt + PIR + PRF, excluding environmental samples from WGS, accessed 18/02/2021). Searches were performed independently in α-, β-, γ-proteobacteria and excluding all three Classes, yielding: 5894 (six iterations), 753 (seven iterations), 1019 (four iterations) and 266 sequences (nine iterations), respectively. This resulted in an initial combined database of 7932 sequences. For FseA matches that contained only the DUF2285 domain, the corresponding DNA locus was inspected for the presence of an upstream PRF site, misannotated start/stop codons and the presence of an upstream DUF6499 domain. DUF6499 domains were identified through the presence of an upstream encoded 'AWEFLRRN’ sequence motif characteristic of the DUF6499 domain. The frameshift site in each DNA locus was edited to produce a open-reading frame and corresponding full-length FseA polypeptide containing both DUF6499 and DUF2285 domains. Shorter QseM-like proteins were distinguished from FseA-like activator protein sequences by their lack of an upstream encoded ‘AWEFLRRN’-like motif. All FseA search matches that retrieved a lone DUF6499 domain were found to be encoded upstream of one of the DUF2285 domain matches identified in the above search and so were removed to avoid duplication. Large protein sequences of more than 400 amino acids were removed, as were sequences that did not contain a distinct ‘AWEFLRRN’ motif after sequence alignment using Clustal Omega. Lastly, sequences containing ambiguous amino acids (i.e. ‘X’) were removed. Overall, ∼59% of sequences of the starting database met the parameters above and coded for an identifiable ‘AWEFLRRN’ motif, making them homologues of FseA. The final sequences were aligned in Clustal Omega with the parameters: clustalo -i Fasta.txt –full -o MSA.fasta –wrap = 10 000 –output-order = tree-order –iterations 6 –max-guidetree-iterations = 6 –max-hmm-iterations = 6.

### Protein structure and interaction modelling

The FseA structure was predicted with trRefineRosetta (14,15) and RoseTTAFold (16) through the Robetta webserver (https://robetta.bakerlab.org/), and with AlphaFold2 (AF2) (17) through ColabFold (18) (https://colab.research.google.com/github/sokrypton/ColabFold/blob/main/AlphaFold2.ipynb). A curated multiple sequence alignment (MSA) supplemented the trRefineRosetta and RoseTTAFold predictions. No template was detected at the time of modelling with trRefineRosetta. The QseM structure was predicted with AF2 through ColabFold. Prediction of coevolving pairs of FseA amino acids (Supplementary Table S4) was carried out using the GREMLIN (19) webserver (http://gremlin.bakerlab.org/) with the curated FseA homologue database using the settings: generate MSA with HHblits with E-value 1E-10 and 0 iterations; filter MSA with coverage 75 and remove gaps 50.

The FseA dimer was modelled in PyMOL based on a AF2-predicted dimer structure that was modelled through ColabFold. Protein docking simulations were performed using the ClusPro 2.0 webserver (https://cluspro.bu.edu/publications.php) (20) with default settings and an N- and C-terminally trimmed version of the FseA AF2 structure (FseA_10-193_) and either the NMR structure of QseM (trimmed to residues 11–83) or the AF2-predicted QseM structure (full length). The FseA-QseM fusion protein (FseA residues 1–195 then QseM residues 1–83) was modelled with AF2 through ColabFold.

### Bacterial two-hybrid assays

*In vivo* QseM-FseA interactions were detected using the Bacteriomatch II Two-Hybrid System (Agilent) as previously described (8), with the following changes: screening medium contained 6.8% (w/v) Na_2_HPO_4_, 3% (w/v) KH_2_PO_4_, 0.05% (w/v) NaCl, 0.1% (w/v) NH_4_Cl; Cm and Tc were added to the final concentration of 25 μg/ml and 12.5 μg/ml, respectively; LB was used as the recovery growth medium after electrotransformation, and no 3-oxo-C6-HSL was added. Protein-protein interaction was detected by growth on selective medium containing 5 mM 3-amino-1,2,4-triazole. Plasmid co-transformation efficiency was determined by growth on nonselective medium. Relative interaction strength was quantified in CFU/ml by the number of colonies growing on selective medium compared to non-selective medium. Biological replicates were performed with three technical replicates.

### NMR spectroscopy

Purified ^15^N/^13^C-labelled 6H-QseM (250 μM) was prepared in 20 mM NaCl, 10 mM NaH_2_PO_4,_ and 10% (v/v) D_2_O for the acquisition of most spectra. For the acquisition of HCCH-TOCSY and ^13^C NOSEY-HSQC spectra, the purified ^15^N/^13^C-labelled 6H-QseM (250 μM) in 20 mM NaCl, 10 mM NaH_2_PO_4_ was lyophilized (Martin Christ, Alpha 3–4 LSCbasic) at room temperature for ∼12 h, then resuspended in the equivalent volume of D_2_O to maintain the buffer concentration. All samples were spiked with 4,4-dimethyl-4-silapentane-1-sulfonic acid (150–300 μM) as a chemical shift standard prior to NMR experiments. Spectra were acquired on a Bruker Avance III 600 or 800 MHz spectrometer at 298 K with a cryogenic TXI probe (600 MHz) or a cryogenic TCI probe (800 MHz).

All NMR data were processed using TopSpin (version 3.5.17) (Bruker) and analysed using the CCPN analysis (version 2.4) (21) software. 81% of expected backbone and 77% of expected side chain ^15^N, ^13^C and ^1^H resonances were assigned. Backbone assignments of 6H-QseM were completed using ^15^N-HSQC, HNCACB, CBCA(CO)NH, and HNCO. Side chain assignments were completed using ^13^C-HSQC, HBHA(CO)NH, ^13^C HCCH-TOCSY, ^13^C HCCH-COSY, HBCBCGCDHD and HBCBCGCDCEHE. Aromatic spin systems were assigned using histidine HSQC, 2D NOESY (150 ms mixing time), 2D TOCSY (100 ms mixing time), and 2D DQFCOSY. TALOS+ was used to predict φ/ψ torsion angle restraints from chemical shift information. ^15^N-edited NOESY-HSQC, ^13^C-edited NOESY-HSQC, and ^13^C-edited aromatic NOESY-HSQC spectra (all recorded with 75 ms mixing time) were used to identify intramolecular protein NOEs through the use of CYANA (version 3.98.5) automated NOE assignment.

CYANA was provided with a list of 680 chemical shift assignments and 535 ^15^N NOESY peaks, 694 ^13^C NOESY peaks, 94 ^13^C aromatic NOESY peaks, and 1488 2D NOESY peaks. Initial structure calculations were performed using a family of 100 structures each running for 10 000 steps of torsion angle dynamics. The 20 lowest energy structures after completion of the CYANA run were then used for subsequent water refinement. Refinement was performed using the RECOORD protocol (22) using 500 annealing runs. The 100 lowest energy structures after annealing were then refined in water and the resulting 20 lowest energy structures were used to form the final family of structures (Supplementary Table S5). The final family structure residues are Ramachandran favoured (76%) and allowed (24%), with one outlier (His33). This set of models has been submitted to the PDB under record number 7UQT.

### Small angle X-ray scattering (SAXS)

Size-exclusion chromatography-coupled synchrotron small angle X-ray scattering (SEC-SY-SAXS) data were collected on purified 6H-QseM using the SAXS/WAXS beamline at the Australian Synchrotron (23,24). Purified 6H-QseM (50 μl at 10 mg/ml) was injected into a Superdex 200 Increase 10/300 GL (GE Healthcare) pre-equilibrated with buffer (10 mM NaH_2_PO_4_, 20 mM NaCl, 2% (v/v) glycerol (pH 7.5)) mounted on a Shimadzu HPLC system with a constant flow rate of 0.25 ml/min at 295 K. A 1-second continuous data-frame was collected using a Pilataus3 S 2M detector at a distance of 1.6 m. Data reduction and background subtraction were carried out using SCATTERBRAIN, and data processed using ATSAS (version 2.8.4) software (25,26). The *P*(*r*), Porod volume and maximum dimension (*D*_max_) were calculated by GNOM (27). The *ab initio* SAXS envelope was generated using DAMMIN (28). SAXS data have been deposited to the SASBDB (29) under the accession code SASDNM8.

### Size exclusion chromatography coupled to multi-angle light scattering (SEC-MALS)

SEC-MALS experiments were carried out using a Superdex Increase 10/300 GL column (GE Healthcare) attached to a Viskotek GPCmax VE 2001 solvent/sample module (Malvern) coupled to a Viskotec 305 TDA detector array (Malvern) at room temperature. Two hundred μl samples of purified 6H-MBP-FseA (1 mg/ml), 6H-QseM (1 mg/ml), a mixture of 6H-MBP-FseA and 6H-QseM (0.5 mg/ml of each protein), or BSA (1 mg/ml) standards in SEC buffer (pH 6.35) were applied to the size-exclusion column pre-equilibrated with SEC buffer at flow rate of 0.2 ml/min. The refractive index, UV absorbance and left and right-angle light scattering of the eluent were constantly monitored. OmniSEC (version 5.10) (Malvern) was used to analyse the SEC profiles and to calculate molecular weight averages and dispersity using calibration settings derived from the average of five BSA standards.

## RESULTS

### FseA binds the IR region of P*rdfS* to activate transcription

**Figure 1.**
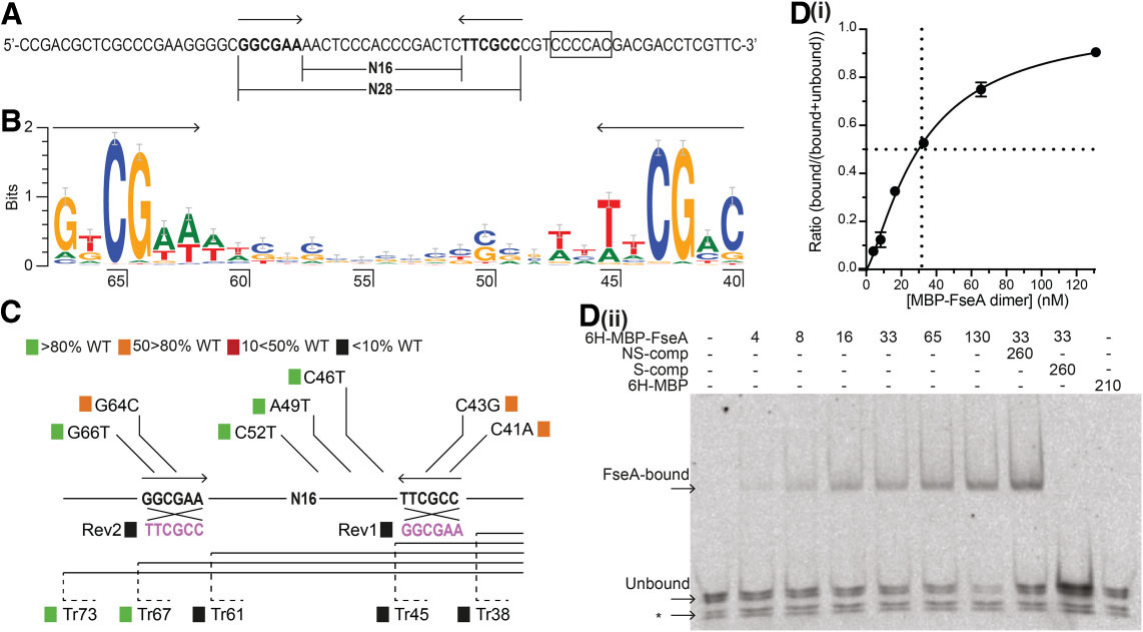
Sequence of the P*rdfS* IR (FseA box) DNA and DNA binding capability of 6H-MBP-FseA. (**A**) Sequence of the P*rdfS* DNA bound by 6H-MBP-FseA. Arrows and bold font denote the IR hexamer sequences of the FseA box, and an open rectangle denotes the P*rdfS* -35 region. The sequence length of the full FseA box and that separating each IR hexamer are denoted. (**B**) Conservation logo from 439 α-proteobacterial P*rdfS* sequences after redundancy filtering (90%). Nucleobase positions represent the length of sequence upstream of the *rdfS* start codon. Arrows denote IR hexamer sequences. The hexamer sequence from nucleobases 62–67 was aligned separately from the upstream sequence to account for skewing of the alignment by spacer lengths of either 15 or 16 base pairs. (**C**) Summary diagram of the activity of P*rdfS* sequence variants in the presence of FseA-6H. Coloured boxes denote activity compared to the wild-type P*rdfS* sequence: green, >80%; orange, 50 > 80%; red, 10 < 50%; black, <10%. Reversed IR hexamer variants, which were constructed and tested individually, are denoted by ‘Rev’ and the modified sequence is shown in purple. Truncations of P*rdfS* are denoted by ‘Tr’. Data used to determine relative P*rdfS* activity of the P*rdfS* variants are presented in Supplementary Figure S1. **(D**) (**i**) *K*_D_ for the 6H-MBP-FseA – P*rdfS* DNA interaction (*n* = 3; error bars show standard error of the mean). (**ii**) EMSA performed with fluorescent-labelled P*rdfS* DNA and 6H-MBP-FseA dimer concentrations of 4–130 nM. NS- and S-comp denote added unlabeled non-specific and specific competitor DNA, respectively. The asterisk denotes fluorescent co-purified P*rdfS* DNA that remains equally unbound at all 6H-MBP-FseA concentrations.

FseA activates transcription downstream of a conserved IR DNA sequence adjacent to the *rdfS* promoter –35 element (9). The IR of P*rdfS* is comprised of two inverted hexamers separated by 16 bp, with each hexamer containing two highly conserved central nucleobases (Figure 1A, B). To establish the role of the IR in P*rdfS* activation, variants of the P*rdfS* sequence were constructed and then cloned upstream of a promoterless *lacZ* gene in plasmid pDSZ*-fseA-6H*. This vector also contained a genetically fused copy of *fseA-6H* (frameshift between *msi172-msi171* removed; sequence encoding 6H added at 3′-end), which is under control of the leaky IPTG-inducible *lac* promoter. Each cloned P*rdfS* variant was then tested for activity in β-galactosidase reporter assays in *M. japonicum* strain R7ANS (which lacks ICE*Ml*Sym^R7A^) in the presence and absence of IPTG-induced *fseA-6H* expression. P*rdfS* variants that contained a truncated IR region showed no β-galactosidase activity, confirming the IR is required for FseA activation of P*rdfS* (Figure 1C). Single nucleotide changes made to either IR hexamer showed either little or no difference (50–80%) in β-galactosidase activity compared to the wild-type sequence, and no difference in activity was observed for single nucleotide changes made to the sequence between the hexamers (Figure 1C). Variants with one of either hexamer sequence in the reverse orientation showed no β-galactosidase activity, indicating the orientation of each IR hexamer was critical for activation (Figure 1C). Together, these results demonstrate that the IR of P*rdfS* facilitates transcriptional activation by FseA and suggested FseA may bind the IR to activate P*rdfS*.

To confirm FseA bound the IR DNA, we expressed FseA as an N-terminal 6H-maltose-binding-protein (MBP) fusion protein. 6H-MBP-FseA was purified from *E*.*coli* by Ni^2+^ affinity chromatography followed by size exclusion chromatography (SEC). Elution of 6H-MBP-FseA in SEC indicated a molecular mass of ∼158 kDa (theoretical monomer mass, ∼75 kDa), suggesting that the protein was a dimer in solution (Supplementary Figure S2A). Although the MBP tag of 6H-MBP-FseA contained a cleavable site, it was not removed because purifications of untagged FseA yielded insoluble aggregates. To investigate whether the MBP tag altered the ability of FseA to activate P*rdfS*, 6H-MBP-FseA was tested for transcriptional activation of P*rdfS*. Sequence encoding 6H-MBP-FseA was cloned under the control of the IPTG-inducible *lac* promoter in plasmid pSDZ-P*rdfS*. In the absence of IPTG induction, the 6H-MBP-FseA plasmid induced β-galactosidase activity to a level ∼3-fold higher than the plasmid carrying *fseA-6H* (Supplementary Figure S3), suggesting that the MBP tag increased the solubility and/or stability of FseA and, therefore, increased the amount of active protein. Both FseA and 6H-MBP-FseA activated P*rdfS* to similar levels when expression was induced with IPTG (Supplementary Figure S3). These results confirmed that the MBP tag did not decrease the transcriptional activity of FseA in the 6H-MBP-FseA fusion.

To assess FseA DNA-binding, electrophoretic mobility shift assays (EMSAs) were performed using purified 6H-MBP-FseA together with a 71-bp dsDNA oligonucleotide containing the conserved IR sequence present upstream of P*rdfS* (Figure 1A). 6H-MBP-FseA produced a single, discrete shift in the migration of the IR DNA and bound with an approximate *K*_D_ of 30 nM (Figure 1D). No shift of the labelled IR DNA was observed when reactions included excess unlabelled IR DNA (S-comp, Figure 1D), whereas the shift was unaffected by the addition of excess DNA amplified from the *fseA* gene (NS-comp, Figure 1D). Thus, FseA specifically bound the IR region upstream of P*rdfS*, which hereafter we refer to as the FseA box.

### The FseA DUF2285 is a variant of the HTH DNA-binding domain

**Figure 2.**
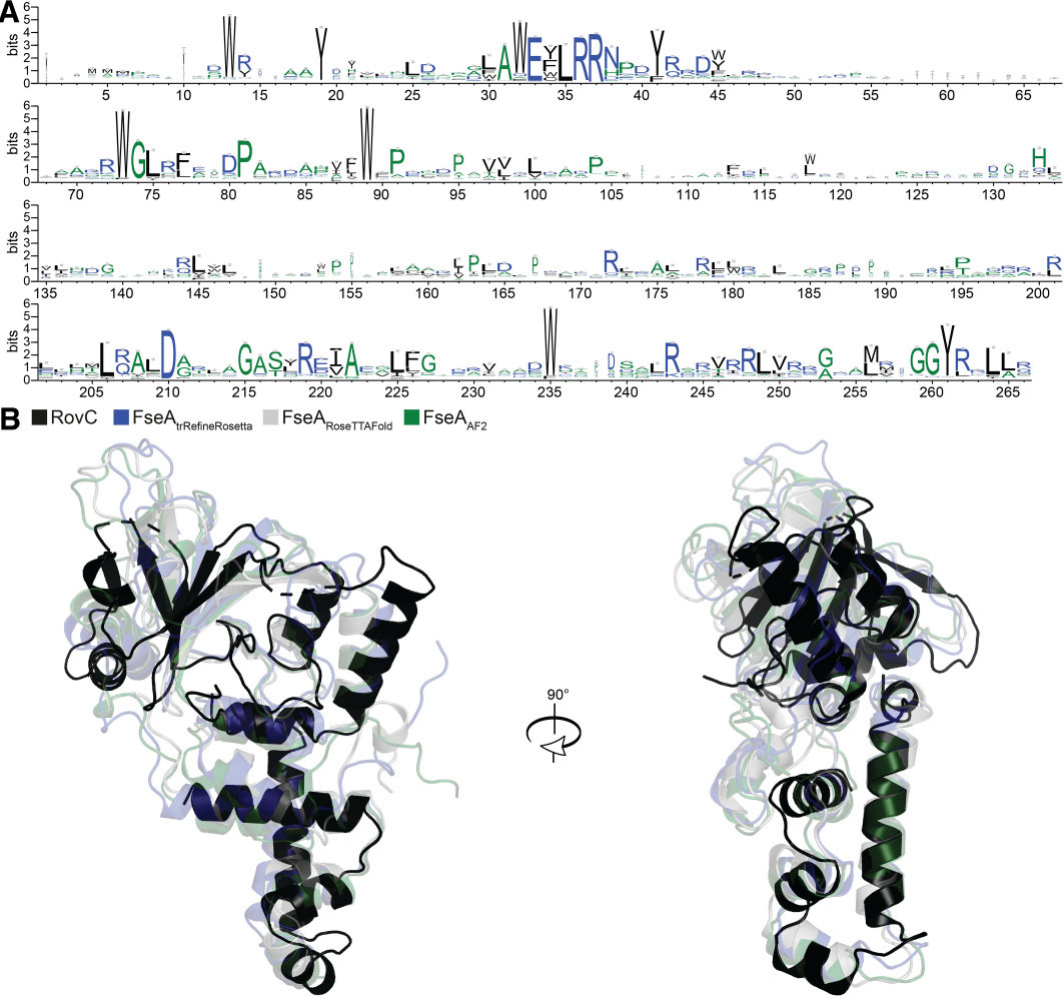
Conservation of amino acids of FseA and comparisons of the RovC crystal structure with structural predictions of FseA. (**A**) Amino-acid conservation logo of FseA generated using the curated FseA database (4709 sequences). Blue, hydrophilic; green, neutral; and black, hydrophobic. The height of each amino acid at each position indicates its conservation. (**B**) Structure prediction of FseA generated with trRefineRosetta (blue), RoseTTAFold (grey) and AF2 (green) aligned with the RovC crystal structure (black).

**Figure 3.**
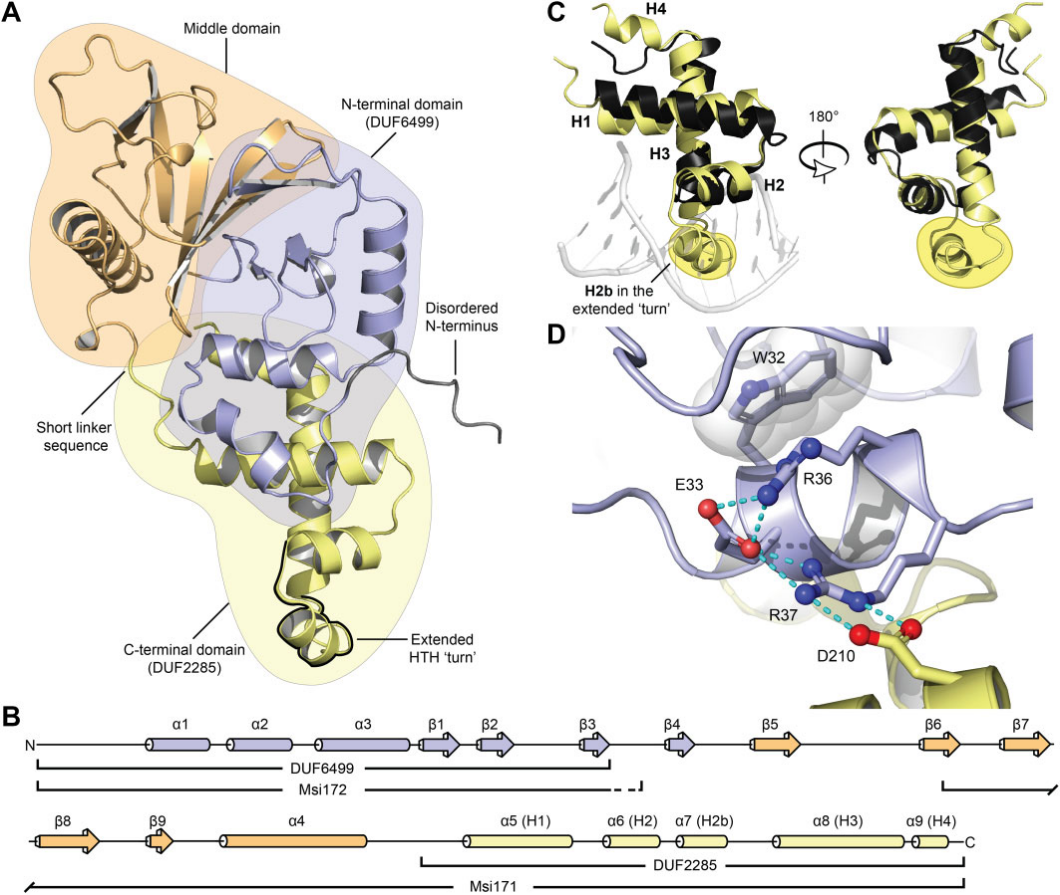
Structural model overview of FseA. (**A**) AF2 FseA structure model coloured by domain: disordered N-terminus, grey; N-terminal domain (containing DUF6499), blue; middle domain, orange; C-terminal domain (containing DUF2285), yellow. The extended ‘turn’ is outlined in black. The five top-ranked models generated by AF2 are shown in Supplementary Figure S4A. (**B**) Secondary structure of the AF2-predicted FseA model (coloured as in A). The DUF6649 and DUF2285 domains are annotated, as well as the Msi172 and Msi171 portions of FseA. The dashed line of the Msi172 annotation represents amino-acid sequence that is not present in FseA (following the +1 PRF). (**C**) Structure alignment of the FseA DUF2285 domain (amino-acids 194–266, yellow) and the crystal structure of the HTH DNA-binding domain of HetR (black) (co-crystalized DNA, grey; PDB 4IZZ). (**D**) Structural model of the FseA DUF2285–DUF6499 interaction showing highly conserved side chains that are identical between FseA and RovC (red spheres, oxygen atoms; blue spheres, nitrogen atoms; cyan dashed lines, hydrogen bonds).

The structure prediction tool trRefineRosetta was used to generate *ab initio* structure predictions for FseA based on coevolving residues inferred from custom sequence alignments. Since *fseA* homologues are often encoded on two separate open reading frames (DUF6499 and DUF2285 that through a +1 PRF generate a single protein), the polypeptide sequences of FseA homologues are frequently misannotated: DUF6499 can be unannotated or annotated with stop codons following the +1 PRF site and DUF2285 domains are generally annotated with incorrect start codons due to the upstream +1 PRF site. Therefore, we manually curated a database of FseA coding sequences, correcting for the + 1 frameshift. We identified 4709 unique FseA homologues from throughout the proteobacteria, ∼61% of which were encoded by two open reading frames separated by the conserved +1 PRF site. The FseA homologues were aligned for use in trRefineRosetta (Figure 2A). The quality score (local distance difference test = 0.75) gave confidence in the overall fold of the predicted FseA structure. Subsequent to this work, the crystal structure of the distantly related RovC protein (PDB 6xz5), which activates expression of a Type VI secretion system in *Yersinia* spp., was published (30) and the structure prediction tools RoseTTAFold and AlphaFold2 (AF2) became available. FseA models obtained with each tool were highly similar to the trRefineRosetta model (Figure 2B), and the AF2 model (Figure 3A) was used for subsequent analyses. The FseA AF2 model exhibited 3.3 Å root-mean-square deviation (RMSD) over 197 residues from the RovC crystal structure, despite FseA and RovC sharing only 22% amino-acid identity over their aligned length.

The FseA model contains three structured domains (demarcated using SWORD (31)), with a disordered sequence at its N-terminus and a short linker-like sequence joining the ‘middle’ and DUF2285 domains (Figure 3A). The N-terminal DUF6499 domain contains three α-helices across residues 10–94, herein termed α1-α3 (Figure 3B). The α2 helix contains the highly conserved ‘AWEFLRRN’ sequence motif (residues 31–38) (Figure 2A), which serves as a central structural component that interconnects the DUF6499 domain and the C-terminal DUF2285 domain: Glu33 positions the side chain of Arg37 such that it interacts with Asp210 of the DUF2285 domain and, together with Arg36, contributes to the placement of the Trp32 side chain that makes hydrophobic contacts in the core of FseA (Figure 3D). α1, which is not present in RovC, makes additional contacts with the DUF2285 domain. The middle domain, which exhibits low amino-acid sequence conservation (Figure 2A), spans residues 95–193 and contains an anti-parallel β-sheet (β-strands 5–9) and an α-helix (α4) that are positioned at the periphery of the structure (Figure 3A, B). The C-terminal DUF2285 domain (residues 194–266), which is highly conserved (Figure 2A), contains five α-helices (Figure 3A, B). The α5 and α9 helices form a cleft that directly interacts with the α2 helix of the DUF6499 domain (Figure 3A). α5 also contacts α1 of the DUF6649 domain through a distinct face.

**Figure 4.**
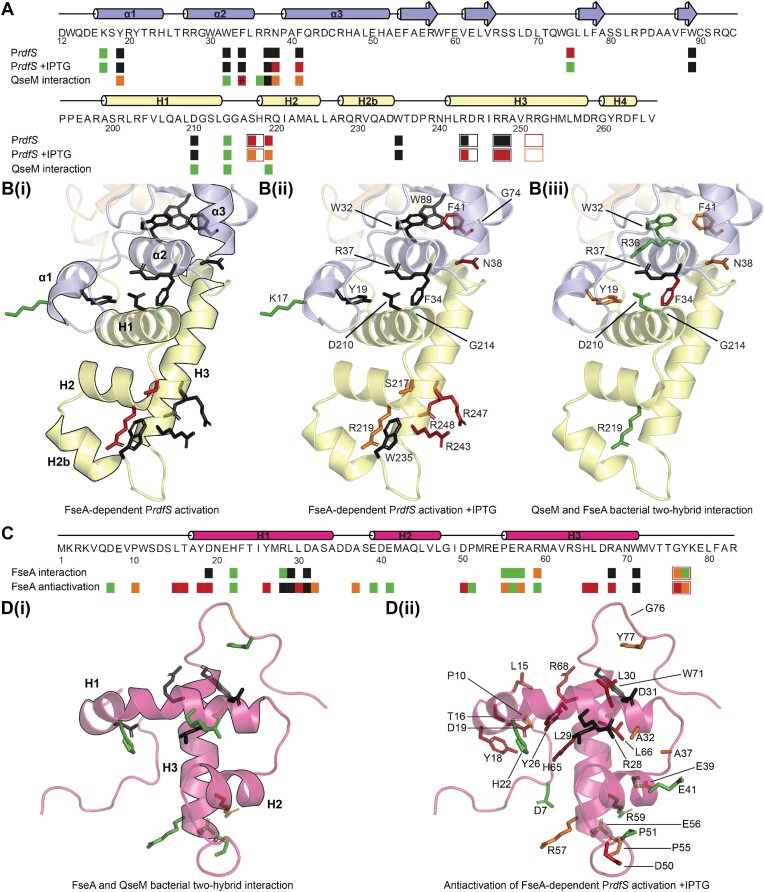
Mutagenesis of FseA and QseM reveals residues required for function. (**A**) P*rdfS* activation of MBP-FseA mutants compared to MBP-FseA, and QseM binding of FseA mutants compared to wild-type FseA are denoted by coloured boxes: green, >90%; orange, 50 > 90%; red, 10 < 50%; black, <10%. Open boxes represent mutants with two substitutions. Note, QseM binding of the F34A FseA mutant is marked by an asterisk because it contained the additional mutation H172Y. Predicted secondary structures of the DUF6499 and DUF2285 domains are shown (DUF6499, blue; DUF2285, yellow). Data used to determine relative activation and binding are presented in Supplementary Figures S6 and S7 respectively. (**B**) Representation of (A) on the FseA model: (**i, ii**), FseA-dependent P*rdfS* activation; (**iii**), QseM bacterial two-hybrid interaction. Mutants with two substitutions are not shown. (**C**) Antiactivation of FseA-6H-dependent P*rdfS* activation and FseA binding of QseM mutants compared to QseM are denoted by coloured boxes as in (A). The secondary structure of QseM is shown in pink. Data used to determine relative binding and antiactivation are presented in Supplementary Figures S7 and S8 respectively. (**D**) Representation of (C) on the QseM NMR structure: (**i**), FseA bacterial two-hybrid interaction; (ii), antiactivation of FseA-6H-dependent P*rdfS* activation. Mutants with two substitutions are not shown.

To test if residues within and around the vicinity of the highly conserved α2 helix were important for FseA function, alanine substitutions were constructed in the α2 region and the resulting alleles were cloned and assayed for their ability to activate P*rdfS*. Almost all mutant proteins were abolished in their ability to activate P*rdfS* (Supplementary Figure S5). Even when the same substitutions were constructed in MBP-tagged FseA and induced with IPTG, the highest activation observed was less than 20% of the wild-type (Figure 4, Supplementary Figure S6). These observations confirm that α2 is critical for function and support its role in maintaining FseA tertiary structure.

DALI (32) was used to compare the predicted FseA DUF2285 structure with structures in the Protein Data Bank (PDB). This revealed that DUF2285 does indeed share structural similarity with DNA-binding HTH domains (Figure 3C), including those present in sigma factors such as SigL (PDB 3HUG (33)), transcriptional activators such as HetR (PDB 4IZZ (34)), and quorum-sensing transcriptional activators such as CviR (PDB 3QP6 (35)). However, the FseA DUF2285 domain deviates significantly from the canonical HTH by containing additional sequence within the turn of the HTH motif, which extends the length of its turn (Figure 3C). To clarify comparisons with the helices of canonical HTH domains (H1, H2, H3) and those of QseM, we refer to FseA helices α5-α9 as H1, H2, H2b, H3 and H4 in following text (Figure 3B, C).

**Figure 5.**
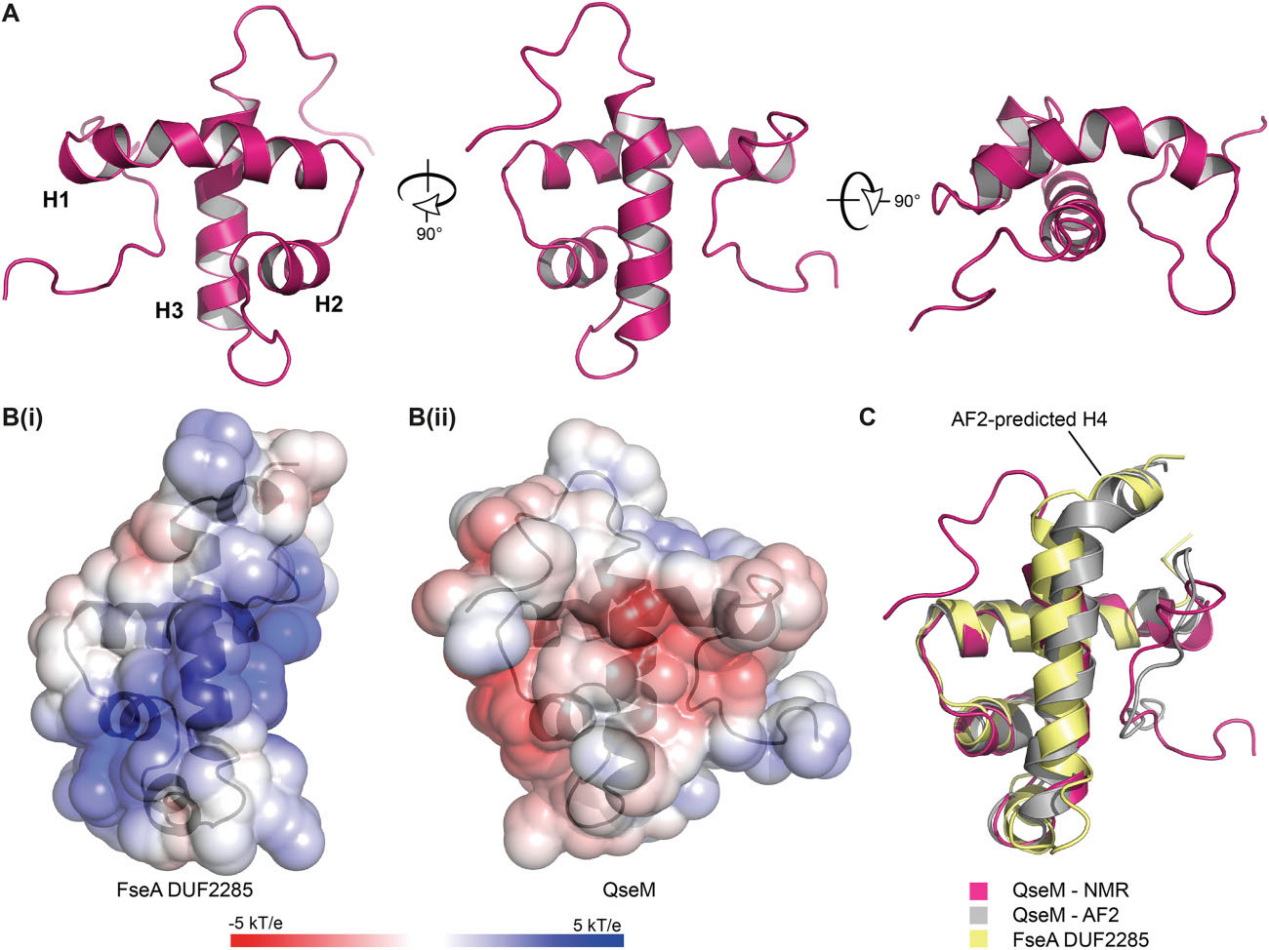
Solution NMR structure of QseM and comparison of the FseA and QseM DUF2285 domains. (**A**) Orthogonal views of the representative NMR structure of QseM (PDB 7UQT) (for NMR ensemble see Supplementary Figure S10A). (**B**) Solvent exposed charge of (**i**) FseA and (**ii**) QseM DUF2285 domains (positive, blue; negative, red). (**C**) Alignment of the QseM AF2 model (grey) and NMR structure (pink) and the DUF2285 domain of the AF2 FseA model (yellow). The five top-ranked models of QseM generated by AF2 are shown in Supplementary Figure S4B.

Within the extended turn between helices H2 and H3 of the FseA DUF2285 domain is a short α-helix (H2b) orientated perpendicular to H1 and H3 (Figure 3C). The C-terminus of H2b places a 79% conserved tryptophan residue Trp235 in a hydrophobic pocket formed by alanine, valine, and leucine residues from H2, H2b and H3, respectively (Supplementary Figure S9). The DUF2285 domain forms an extensive positively charged surface (Figure 5B(i)), with a net positive charge of +7 (15–6–1–1; Arg-Asp-Glu-COO- (C-terminus)), which is consistent with a role in DNA binding (36).

To test if the solvent-exposed positively charged residues in the FseA DUF2285 domain were required for transcriptional activation by FseA, alanine substitution mutants of 6H-MBP-FseA were tested *in vivo* for their ability to activate expression from P*rdfS*. These mutants were tested in the 6H-MBP fusion only so that any destabilising effects of the mutations could be minimised by the added stability/solubility of MBP. Singly substituted proteins with R243A, R247A and R248A showed P*rdfS* transcriptional activation of less than 10% of wild-type 6H-MBP-FseA without, and 40% with, IPTG-induced 6H-MBP-FseA expression (Figure 4A, B). 6H-MBP-FseA containing a double substitution, R247A and R248A, showed no transcriptional activation from P*rdfS* even under IPTG-induced conditions (Figure 4A). Purified 6H-MBP-FseA_R247A–R248A_ exhibited an apparent molecular mass of ∼160 kDa in SEC experiments (Supplementary Figure S2B), indicating oligomerisation was unaffected by the substitutions. EMSAs carried out using 6H-MBP-FseA_R247A–R248A_ revealed that the mutated protein exhibited greatly reduced binding affinity to the FseA box, with 3 μM protein concentration only shifting ∼60% of the DNA (Supplementary Figure S11). We next investigated the Trp235 residue present at the end of H2b and central to the hydrophobic pocket formed between H2, H2b and H3. A W235A substitution abolished transcriptional activation by FseA *in vivo* (Figure 4A, B), consistent with its high conservation in FseA homologues and possible key role in the hydrophobic pocket of the domain. In summary, purified FseA is a dimeric DNA-binding protein that interacts with DNA containing the FseA box that is upstream of P*rdfS*, with positively charged residues in the HTH-like DUF2285 domain being required for DNA binding.

### QseM is a HTH variant akin to the FseA DUF2285 domain but lacks a positively charged surface and H2b

The QseM polypeptide contains a DUF2285 domain with 18% amino-acid identity to that of the FseA DUF2285 domain. To gain insight into the structure of QseM, we undertook solution small-angle X-ray scattering (SAXS) on purified 6H-QseM. The data (Supplementary Figure S12) indicated 6H-QseM was an ellipsoidal globular monomer with a molecular mass in solution of 10 400 Da, close to the theoretical mass of 10 960 Da. The molecular dimensions (*R*_g_ 16 Å and *D*_max_ 57 Å) and the shape of the pair-distribution function were commensurate with a prolate ellipsoid, and Kratky analysis indicated a substantially ordered structure.

We next determined the three-dimensional structure of 6H-QseM using solution NMR spectroscopy. All or most atoms of the residues Gln6-Val9, Ser12, Leu15-Arg53, Pro55-Trp71 and Leu80-Arg83, encompassing 78% of the QseM sequence, were assigned. Only the polypeptide backbone atoms were assigned for the residues Val5, Trp11, Glu54 and Met72. No atoms of the residues Ser1-Lys4, Asp7, Glu8, Pro10, Asp13, Ser14 and Val73-Lys79 were assigned. Residues 6–74 of QseM form an ordered trihelical arrangement, whereas both termini (residues 1–5 and 75–83) are fully or partially disordered. The ensemble of the 20 lowest energy models overlaid with an RMSD of 0.36 Å across the backbone heavy atoms of residues 6–72 (Supplementary Figure S10A). CRYSOL (25) comparison of the NMR structure to the SAXS data for QseM yields a very good fit (χ^2^ = 0.30, where 0.25 represents an ideal fit for Australian Synchrotron data). Taken together, the solution structure measurements both support the observation that QseM is a globular monomer in solution. The NMR structure and data have been deposited in the PDB with the code 7UQT. A single representative structure was used for further analysis (Figure 5A).

6H-QseM exhibits three α-helices (H1, residues 17–34; H2, residues 39–46; H3, residues 55–71) with the H3 helix forming the backbone of the structure that the other two helices cross at close to 90°, creating a hydrophobic core (Figure 5A). The H1 helix is curved along its length with an overall deviation of around 40 degrees, allowing it to partially wrap around H3. The structure of QseM is akin to that of the FseA DUF2285 domain, as it is also comprised of a HTH-like fold that contains an extended turn between H2 and H3 (Supplementary Figure S10C). Compared to the FseA DUF2285, the solution structure of QseM lacks H2b and H4. While lacking structure in the H4 region, this region of QseM contains the highly conserved GY sequence motif at the turn directly preceding H4 that is present in other DUF2285 domains (Figure 2A). QseM is more compact than the FseA DUF2285 due to the bend in H1. Notably, QseM lacks the extensive positively charged surface present in the FseA DUF2285 that is involved in DNA binding (Figure 5B). We also generated models of QseM using AF2. The AF2 QseM model agreed closely with the NMR structure (RMSD 1.71 Å over residues 15–71) (Figure 5C), except that the predictions indicated the presence of the H4 helix at the C-terminus of QseM. The AF2 prediction, together with the dearth of chemical shift assignments in this region (e.g. for residues Thr74-Gly76, Lys78 and Glu79) and the presence of a corresponding H4 helix in FseA and RovC, suggest the existence of a conformational exchange process at the C-terminus of QseM, perhaps involving the folding-unfolding of a short H4 helix.

### QseM is unlikely to bind DNA and is unable to inhibit FseA *in vitro*

The lack of the extensive positively charged surface on QseM suggested that it may lack DNA-binding activity. Indeed, we observed no binding by QseM to the FseA box in EMSAs (Supplementary Figure S13A). We also failed to observe a complex between 6H-QseM and dimeric 6H-MBP-FseA in SEC experiments and did not observe any difference in 6H-MBP-FseA binding to the FseA box when co-incubated with excess 6H-QseM in EMSAs (Supplementary Figure S13), despite QseM binding Msi172 (composed of DUF6499; Figure 3B) and FseA in bacterial two-hybrid assays. This suggests that either QseM cannot bind mature 6H-MBP-tagged FseA dimers, or that the 6H and 6H-MBP tags interfered with binding *in vitro*. To investigate whether the protein tags interfered with binding, a plasmid expressing 6H-QseM from the constitutive *nptII* promoter was introduced into R7ANS carrying plasmid pSDZ-*fseA*-6H-P*rdfS*. Expression of 6H-QseM completely blocked FseA-6H-dependent activation of P*rdfS* both in the presence and absence of IPTG induction, suggesting that the 6H-tag of QseM or FseA did not interfere with their interaction (Supplementary Figure S14). Likewise, the activity of 6H-MBP-FseA was completely blocked by 6H-QseM in the absence of IPTG and partially reduced when induced with IPTG (Supplementary Figure S14), confirming that 6H-QseM was able to inhibit 6H-MBP-FseA *in vivo*. Therefore the 6H and 6H-MBP tags do not prevent QseM-FseA interactions *in vivo*. Taken together, the lack of QseM DNA-binding in EMSAs and a paucity of surface-exposed positively charged amino acids on QseM H3 suggests that QseM antiactivation of FseA does not require interaction with DNA. Also, while tagged FseA and QseM appear to interact *in vivo*, no interaction was observed with tagged homomeric FseA and QseM *in vitro*, indicating that QseM cannot access its binding site in mature FseA dimers.

### QseM H2 and H3 are not required for binding or antiactivation of FseA, while H1 and the C-terminus are essential

To identify regions of QseM key to its interaction with FseA *in vivo*, alanine-scanning mutagenesis was carried out for QseM. Alanine substitution variants were tested for their ability to inhibit FseA-6H activation of P*rdfS* using β-galactosidase assays, and a selection of these variants were tested further for interactions with FseA in bacterial two-hybrid assays. Given that QseM lacks a positively charged surface in the likely DNA-binding region, we wondered if exposed residues surrounding the H2-H3 region were important for FseA antiactivation. QseM variants carrying alanine substitutions of solvent-exposed charged residues in H2 and at the base of H3 (Glu41, Glu56, Arg57 and Arg59), corresponding with residues in FseA required for activation of P*rdfS* and DNA binding, were therefore constructed. The QseM mutants exhibited wild-type-like FseA antiactivation and variants tested in bacterial two-hybrid assays (Glu56, Arg57 and Arg59) interacted similarly to wild-type 6H-QseM (Figure 4C, D). These results suggest antiactivation of FseA did not involve the H2-H3 region of QseM, consistent with the QseM H2-H3 quasi-DNA-binding region not being required for antiactivation of FseA-dependent transcription.

Mutants of QseM containing alanine substitutions in residues Tyr18, Asp19, Tyr26, Leu29, Leu30, His65 and Leu66, which form interhelical contacts within the QseM structure, were each reduced in their antiactivation of FseA (Figure 4C, D), consistent with a role in maintaining QseM structure. In contrast, mutants with alanine substitutions in exposed residues bordering the structural core (Asp7, His22, Glu39, Glu41, Pro51, Glu56 and Arg59) were similar to wild-type QseM in their ability to repress transcriptional activation by FseA-6H (Figure 4C, D). However, alanine substitutions of solvent-exposed residues Arg28 and Asp31 of H1 and C-terminal residues Arg68, Gly70 and Trp71 impaired or abolished antiactivation of FseA-6H (Figure 4C, D). Bacterial two-hybrid assays revealed that these same substitutions (apart from R28A) also reduced QseM binding to FseA (Figure 4C, D). These results suggest that both the solvent-exposed side of H1 and the very C-terminus of QseM (corresponding to H4 of the DUF2285 domain in FseA) play an essential role in the binding and antiactivation of FseA.

### QseM binds the DUF6499 domain of FseA

Previously reported bacterial two-hybrid assays indicated the N-terminal portion of FseA containing DUF6499 was sufficient for interaction with QseM (9). To further delineate regions of FseA required for QseM interaction, we constructed a series of FseA N- and C-terminal truncations (Supplementary Figure S15). Truncated FseA lacking both the middle and DUF2285 domains (FseA_1–85_) interacted with QseM as strongly as wild-type FseA in bacterial two-hybrid assays (Supplementary Figure S7A), confirming our previous observations. Further truncation (FseA_1–55_) reduced QseM interaction to ∼40% the strength of wild-type, suggesting that this truncation bordered residues that are critical for QseM binding (Supplementary Figure S7A). Truncation of the N-terminal 15 amino acids of FseA (FseA_15–266_) had no effect on QseM binding, while truncations FseA_20–266_ and FseA_25–266_ exhibited severely reduced QseM interaction (Supplementary Figure S7A). This delineated FseA amino-acids 15–55, which contain helices α1 and α2, as being necessary and sufficient for QseM binding.

### QseM may mimic the FseA DUF2285 domain to bind the FseA α2 helix of DUF6499

The structural similarity of the QseM and FseA DUF2285 domains led us to suspect that QseM binds the DUF6499 domain of FseA by mimicking the interdomain amino-acid contacts made by the FseA DUF2285 domain. In doing so, QseM may displace the FseA DUF2285 domain and thus deactivate FseA. The α2 helix of the DUF6499 domain of FseA exhibits high sequence conservation amongst FseA homologues (Figure 2A) and, together with α1, makes the majority of interdomain contacts formed between the FseA N-terminus and the DUF2285 domain in FseA structure predictions.

The DUF6499 α2 helix forms a hydrophobic pocket with helices H1 and H4 of the DUF2285 domain. The side chain of Phe34 of α2 is positioned in the centre of this hydrophobic pocket, whilst the nearby Trp32 and Arg36 residues are predicted to protrude away in the opposite direction (Figure 4B(iii)). We hypothesised that a Phe34 alanine substitution mutant might show reduced QseM interaction due to a loss of hydrophobic contacts with QseM, while mutants carrying alanine substitutions of Trp32 and Arg36 might show wild-type-like QseM interactions. Indeed, bacterial two-hybrid assays revealed reduced interaction of the F34A FseA mutant to QseM, whilst mutant FseA proteins carrying alanine substitutions of Trp32 or Arg36 exhibited wild-type-like QseM interactions (Figure 4A, B). Together, these observations support the role of a hydrophobic pocket forming between the FseA DUF6499 domain and QseM during the QseM-FseA binding interaction and confirm that FseA residues Trp32 and Arg36 are not involved in the interaction with QseM.

The highly conserved DUF6499 α2 residue Arg37 is strongly co-evolving with Asp210 (in DUF2285 H1) in FseA homologues (GREMLIN; Supplementary Table S4) and the two residues are in contact in all FseA structure predictions and the RovC crystal structure (RovC Arg16 and Asp193) (Figure 3D). Alanine substitutions in either FseA Arg37 or Asp210 abolished the ability of the mutant proteins to activate transcription from P*rdfS* (Figure 4A, B), supporting the importance of this contact in the activity of FseA. The QseM residue Asp31 of H1, which abolishes QseM activity when mutated (Figure 4C, D), is reciprocal to Asp210 of the FseA DUF2285 domain. We hypothesised that Asp31 of QseM might interact with Arg37 of FseA. Indeed, FseA carrying an alanine substitution in the Arg37 residue showed near zero interaction with QseM in bacterial two-hybrid assays (Figure 4A, B), making it probable that a salt-bridge forms between Arg37 of FseA and Asp31 of QseM. Together, these results are consistent with a model wherein the FseA DUF6499 α2 helix interacts with QseM H1 and C-terminus in a mechanism that is analogous to its interaction with the FseA DUF2285.

To visualize the potential interaction interface between QseM and the FseA DUF6499 domain, AF2 was used to produce a model of the FseA N-terminal domain fused to QseM (FseA residues 1–198 and QseM residues 17–83), such that the QseM DUF2285 domain replaced that of FseA. The resulting AF2 model placed QseM helices H1 and H4 cradling the FseA α2 helix as expected (Supplementary Figure S16A). This model placed QseM Asp31 (corresponding with Asp210 in the FseA DUF2285 domain) in contact with FseA Arg37, consistent with our prediction that these residues form a salt-bridge during the interaction of QseM and FseA. The AF2 model also placed H1 residues of QseM in contact with α1 of DUF6499. Residues in this interface notable for their conservation include Arg28 of QseM and Tyr19 of FseA. Alanine substitution of QseM Arg28 abolished repression of FseA activation of P*rdfS* but did not reduce binding to FseA in bacterial two-hybrid assays (Figure 4C, D), and alanine substitution of Tyr19 of FseA showed a minor decrease in binding to QseM (Figure 4A, B). These data suggest the interaction of QseM with FseA α1 is not essential for FseA-QseM binding but is required for a productive antiactivation interaction with FseA.

Rigid-body docking simulations (ClusPro) were performed with a truncated FseA structure (amino acids 9–184; N-terminus trimmed, DUF2285 domain removed) and either the QseM NMR structure (amino acids 11–83) or AF2 model. Interestingly, docking simulations of truncated FseA with the AF2-predicted QseM model, which contains H4, closely approximated the FseA DUF2285-DUF6499 interaction (Supplementary Figure S16B). The H4 helix is present in the C-terminus of all DUF2285 domain structure predictions and in RovC, whereas the C-terminus of the QseM NMR structure is disordered. It is possible that the C-terminus of QseM forms a more structured helical (H4) region upon FseA binding. QseM residues directly preceding the putative fourth helix, such as Arg68 and Trp71, are highly conserved amongst QseM homologues and are critical for FseA antiactivation and binding (Figure 4C, D), supporting a role for this region in the QseM-FseA interaction.

In summary, we confirmed that solvent-exposed QseM residues of H1 and the C-terminus, are required for effective binding and activation of FseA and that residues in the FseA DUF6499 α2 helix are involved in binding QseM. Together, these observations suggest that QseM likely makes the same contacts with the DUF6499 domain as the FseA DUF2285 domain does.

## DISCUSSION

The DUF2285-domain proteins FseA and QseM are master regulators of ICE*Ml*Sym^R7A^ transfer and likely control the transfer of numerous mobile genetic elements present throughout the proteobacteria. In this work, we show that the DUF2285 domain represents a previously unrecognized variant of the HTH motif. Purified 6H-MBP-FseA is dimeric in solution and binds the FseA box upstream of P*rdfS*, consistent with the function of FseA as a transcriptional activator. Structural predictions followed by mutational analyses revealed that FseA likely adopts a similar fold to the *Yersinia pestis* transcriptional activator RovC, and that the FseA DUF2285 domain exhibits an extensive positively charged surface that is critical for its transcriptional activation and DNA binding functions. The NMR structure of the antiactivator QseM revealed that it is comprised of HTH-like domain similar to the FseA DUF2285 domain, with disordered N- and C-termini. Despite the similarity of QseM to the DUF2285 of FseA, QseM has an overall negatively charged surface and is unable to bind the FseA box. Transcriptional activation and bacterial two-hybrid assays carried out with mutated and truncated FseA/QseM proteins revealed QseM achieves antiactivation of FseA by binding its N-terminal DUF6499 domain. Both FseA and QseM DUF2285 domains are predicted to similarly contact the highly conserved α2 helix of the DUF6499 domain. FseA substitution mutants in either the DUF2285 or DUF6499 domain that were predicted to disrupt this interaction destroyed the ability of the mutant FseA protein to activate transcription, while corresponding DUF2285 substitutions in QseM prevented it from antiactivating FseA. Therefore, DUF6499 constitutes a critical structural component of FseA and represents the binding target of QseM.

The archetypical HTH domain forms a trihelical arrangement in which H2 and H3 are separated by a short, almost universally conserved ‘turn’ that is poor at tolerating insertions (36). In contrast, both FseA and QseM DUF2285 domains contain a substantial insertion in the turn which, in the case of FseA, includes an additional short helix, termed here H2b. Other HTH-domain proteins with extended turn motifs between H2 and H3 include the chitin sensor protein ChiS from *Vibrio cholerae* (37), and the Q antiterminator of lambdoid phages (38). The extended H2-H3 motifs of Q and ChiS function to enhance DNA interactions, making it possible that residues in the extended-turn of the FseA DUF2285 make DNA interactions. When compared to DNA-bound HTH domains that were detected in DALI searches, the FseA H2b appears to clash with DNA bases (Supplementary Figure S17A); however, comparisons to some HTHs not detected in DALI searches, such as the DNA-bound winged-HTH of the transcriptional response regulator KdpE (PDB 4KNY (39)), placed H2b in a position that does not clash with DNA and, importantly, placed key DNA-binding H3 residues in the DNA major groove (Supplementary Figure S17B). Taken together, it is possible that H2b residues make DNA contacts that stabilize DUF2285-DNA binding and help to position key H3 residues in proximity of DNA major groove nucleobases.

**Figure 6.**
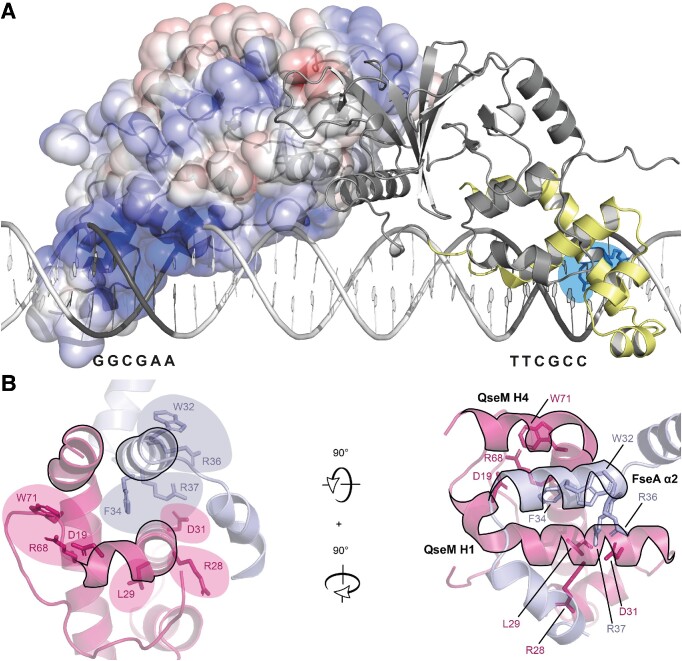
Model of FseA binding the FseA-box IR and QseM interaction with DUF6499 domain of FseA. (**A**) Model of an FseA dimer binding the FseA box (as B-form DNA) through its DUF2285 domains (pale yellow). The right-hand subunit highlights the predicted positions of Arg247 and Arg248 in blue, while the left-hand subunit shows solvent-accessible electrostatic surface coloured by electrostatic potential (positive, blue; negative, red). IR hexamer base-pairs are coloured dark grey on the DNA model. (**B**) FseA DUF6499 and QseM DUF2285 of the AF2-folded QseM-FseA fusion protein (Supplementary Figure S16). Experimentally determined residue side chains in the QseM-FseA interaction model that support the model when mutated to alanine are shown as sticks and labelled. FseA α1–3 and its residue side chains are coloured blue and QseM pink. FseA α2 and QseM helices H1 and H4 are outlined in black.

The FseA box is notable because of the distance between the centre of each IR hexamer, which spans approximately two DNA turns (22 base pairs). We observed no evidence of additional binding events in our EMSAs that might suggest sequential binding of two sites by separate molecules. Therefore, individual DUF2285 domains in a single FseA dimer are likely to bind a single hexamer sequence. Modelling of FseA dimers based on an AF2-predicted homodimeric FseA model produced a plausible protein-DNA complex with individual DUF2285 domain H3s positioned in proximity of a hexamer of the FseA-box IR (Figure 6A). Overall, we propose that the extended-turn and extensive positively charged surface of the DUF2285 domain function to stabilize FseA dimer binding over the span of the 28-basepair FseA box.

FseA homologues are widespread throughout proteobacteria. Their representation and, relatedly, the coexistence of DUF2285 and DUF6499 domains is notably underestimated due to the presence of a +1 PRF. In our curated dataset, around 61% of FseA homologues require a +1 PRF, and are thus not annotated as intact proteins by high-throughput genome annotation pipelines. We have not detected any case where a bona fide DUF6499 domain is not adjoined to a DUF2285 domain. There is a knock-on effect of this segregation of apparent open reading frames in protein domain prediction tools (e.g. Pfam (40)), which may be unable to detect structural motifs in what are, in practical terms, highly conserved single proteins. It is also possible that the DUF2285 domain was not initially identified as a variant of the HTH domain because of the unusual sequence distance between H2 and H3. Having shown here that the DUF2285 domain is a novel HTH domain variant, we propose that DUF2285 should be included in the Pfam HTH superfamily and that FseA-like variants be named to Helix-extended-Turn-Helix domain-containing proteins (HeTH) to better distinguish this family of proteins. Overall, these observations suggest that other DUFs may be variants of well-characterized domains and, with developments in *ab initio* contact and structure prediction, may lead to other DUFs to be more readily linked to existing structural families.

QseM likely evolved from a gene duplication of an FseA ancestor that retained DUF6499 binding while losing the ability to bind DNA and homo-oligomerize. While most HTH domains bind to DNA, some have evolved to mediate protein-protein interactions or make structural units in enzymatic complexes (41–43). We propose a model in which QseM prevents FseA from adopting its native conformation required for transcriptional activation by forming a QseM-DUF2285 - FseA-DUF6499 heterodimer, wherein QseM mimics the FseA DUF2285 domain (Figure 6B).

Protein structure prediction and comparison with the RovC crystal structure suggest that the DUF2285 domain C-terminus contains a short helix (H4), which is disordered in the QseM NMR structure. Disorder-to-order transitions are a well-described feature of regulatory networks. For example, the interaction domains of the p160 and p300 hormone receptor coactivators are intrinsically unstructured; however, upon interaction they form a structured trihelical arrangement (44). It is thus likely that the QseM C-terminus is disordered in isolation but adopts an α-helical structure when interacting with FseA. The inherent flexibility of this region may allow it to contribute to more diverse protein-protein interactions, such as the currently structurally uncharacterized interaction between QseM and the unrelated quorum-sensing transcriptional activator, TraR.

QseM may bind and sequester FseA while FseA is partially unfolded, or prior to the formation of transcriptionally active dimers. This suggestion is based on the preliminary observations that purified 6H-QseM does not bind dimeric 6H-MBP-FseA or inhibit its DNA-binding activity *in vitro*, despite 6H-QseM being capable of antiactivating 6H-MBP-FseA *in vivo*. It is also possible that *in vivo* QseM acts early in the life of FseA, perhaps binding to the DUF6499 domain during its translation. The positioning of the PRF that results in the fusion of the DUF6499 domain to the remainder of FseA is curious because the highly conserved adjacent WGL sequence is encoded by a ribosomal binding site-like mRNA sequence (UGGGGG). This sequence may stall translation, allowing the nascent FseA DUF6499 domain to bind to QseM prior to translation of the remainder of FseA. Given that overexpression of FseA is bacteriostatic in *M. japonicum* R7A (mediated through induction of P*rdfS* (6,7)), acute negative regulation of functional FseA is essential for R7A cells to survive. This selection pressure has likely led to the evolution of the observed multi-layered transcriptional, translational, and post-translational repression of FseA through QseM antiactivation of TraR, the +1 PRF and QseM antiactivation of FseA, respectively.

In summary, we show that the DUF2285 and DUF6499 domains form an interacting pair. While both domains are commonly found within a single FseA-family protein that is capable of transcriptional activation, ‘loss of function’ QseM-family variants containing only the DUF2285 domain are capable of binding and inhibiting the conjoined activator. The detection and determination of function of the DUF2285 and DUF6499 domains had been obscured in genomic analyses but has been resolved here by a comprehensive structure-function analysis.

## Supplementary Material

gkad457_Supplemental_File

## Data Availability

The QseM structure and data have been deposited in the PDB with the code 7UQT. SAXS data have been deposited to the SASBDB under the accession code SASDNM8.
