# Supplementary material for: DUF2285 is a novel helix-turn-helix domain variant that orchestrates both activation and antiactivation of conjugative element transfer in proteobacteria
Source: Nucleic Acids Res. 2023 May 29;51(13):6841–56. doi: 10.1093/nar/gkad457 (PMC10359603; doi:10.1093/nar/gkad457)
Supplement: gkad457_Supplemental_File [file gkad457_supplemental_file.pdf]

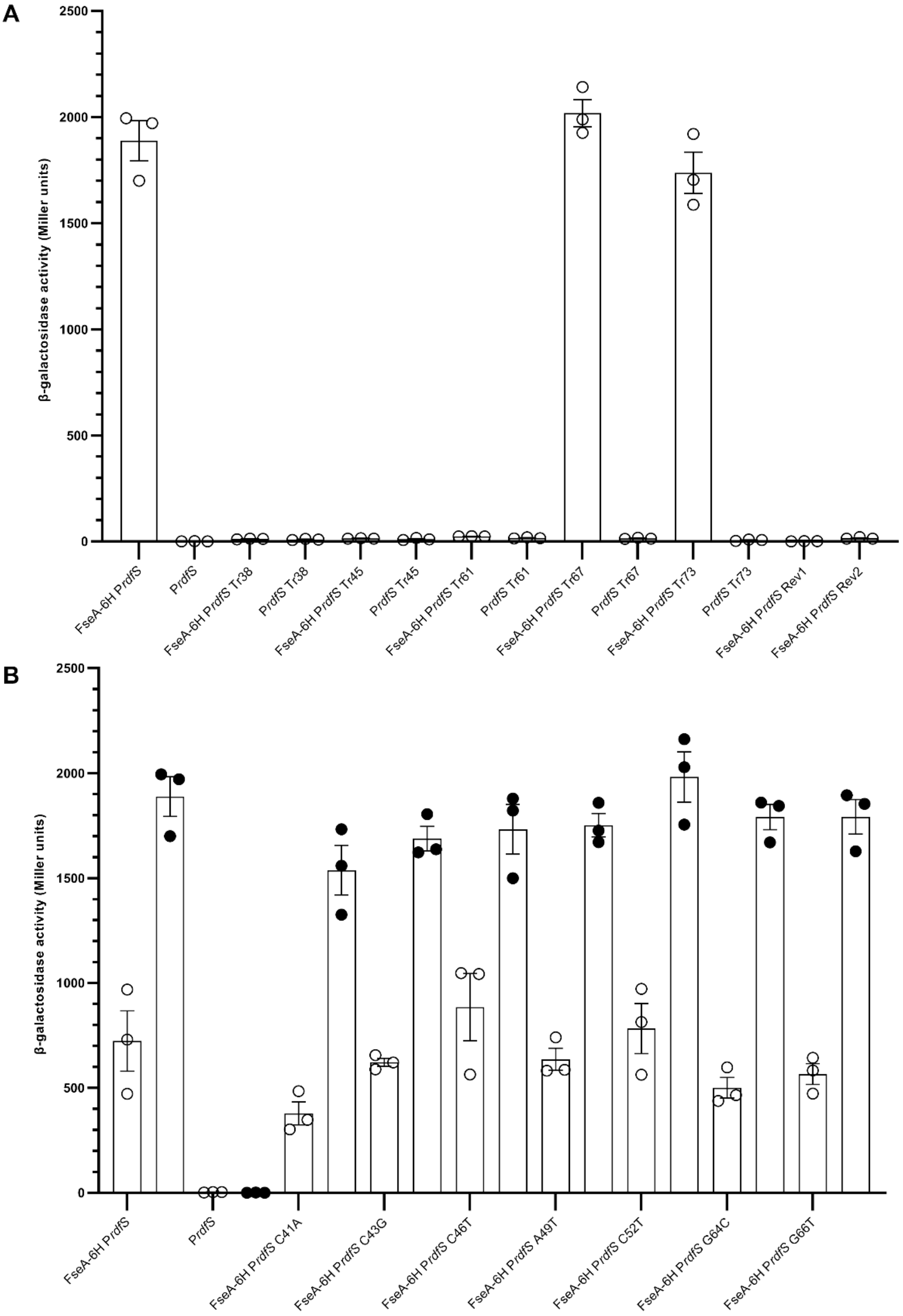

Figure S1. Mutagenesis of P*rdfS* reveals the IR sequence motif is required for FseA-dependent activation. Mean P*rdfS* activation is represented by columns, with circles denoting values from biological replicate assays (each an average of two technical replicates). Error bars show the standard error of the mean (SEM). All samples in (**A**) were IPTG-induced for increased *lac* promoter expression. 'Tr' denotes truncated P*rdfS* variants; and 'Rev', reversed hexamer variants. Labels on the x-axis in (**B**) span two groups (uninduced, open circles; IPTG-induced *lac* promoter expression, filled circles). Values were normalised by the proportional difference of a single representative experiment's positive control and the respective experiment's positive control (performed independently in (**A**) and (**B**), and independently for uninduced and induced samples in (**B**)).

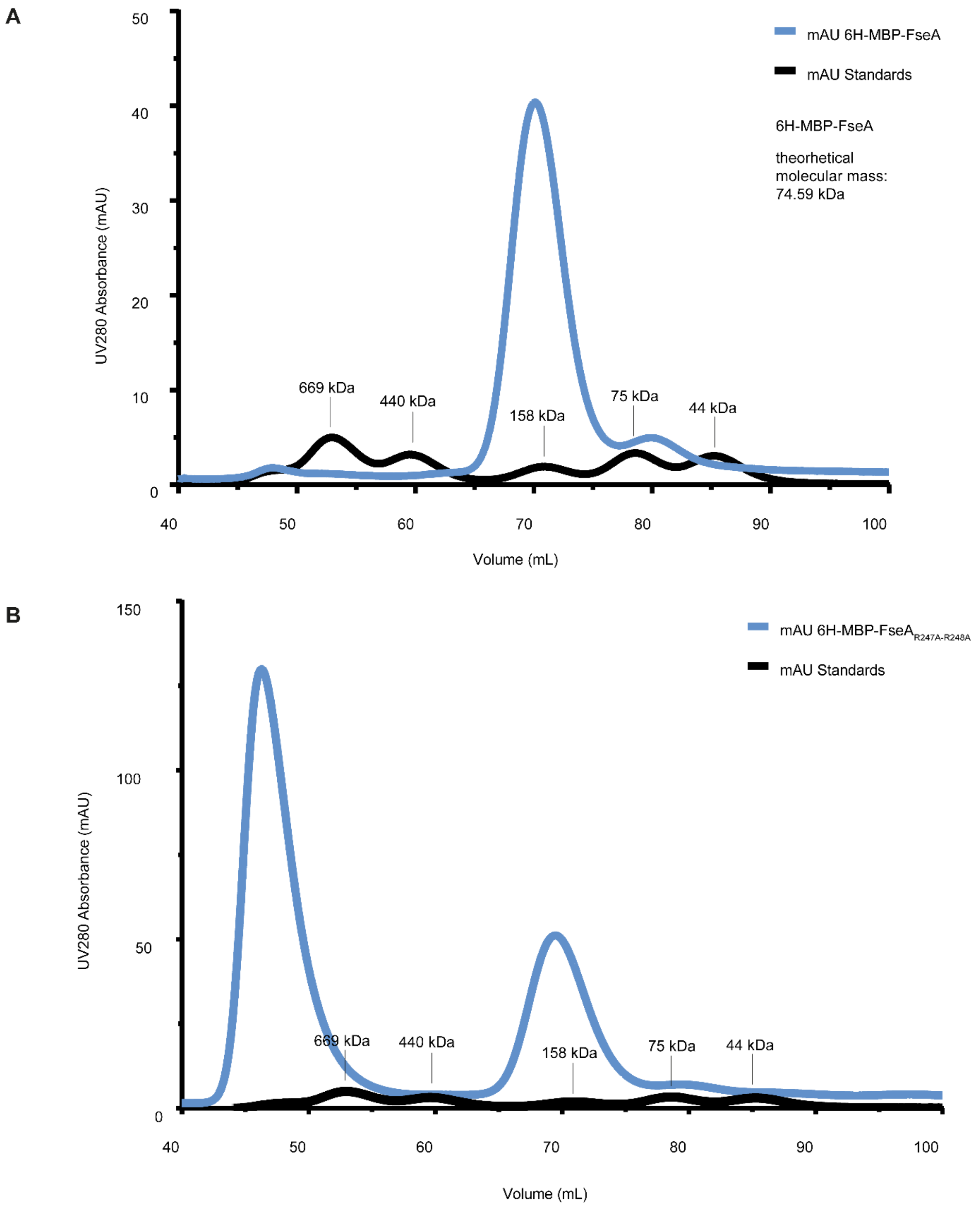

Figure S2. 6H-MBP-FseA and 6H-MBP-FseA$_{R247A-R248A}$ are both dimeric in solution. Chromatography traces of the SEC purification of 6H-MBP-FseA and 6H-MBP-FseA$_{R247A-R248A}$ reveal that both proteins form homodimers. UV280 absorbance of (**A**) 6H-MBP-FseA and (**B**) 6H-MBP-FseA$_{R247A-R248A}$ are shown in blue, while the absorbance of size standards (GE Gel Filtration Calibration Kit high molecular weight, run separately on the same system set-up) are in black.

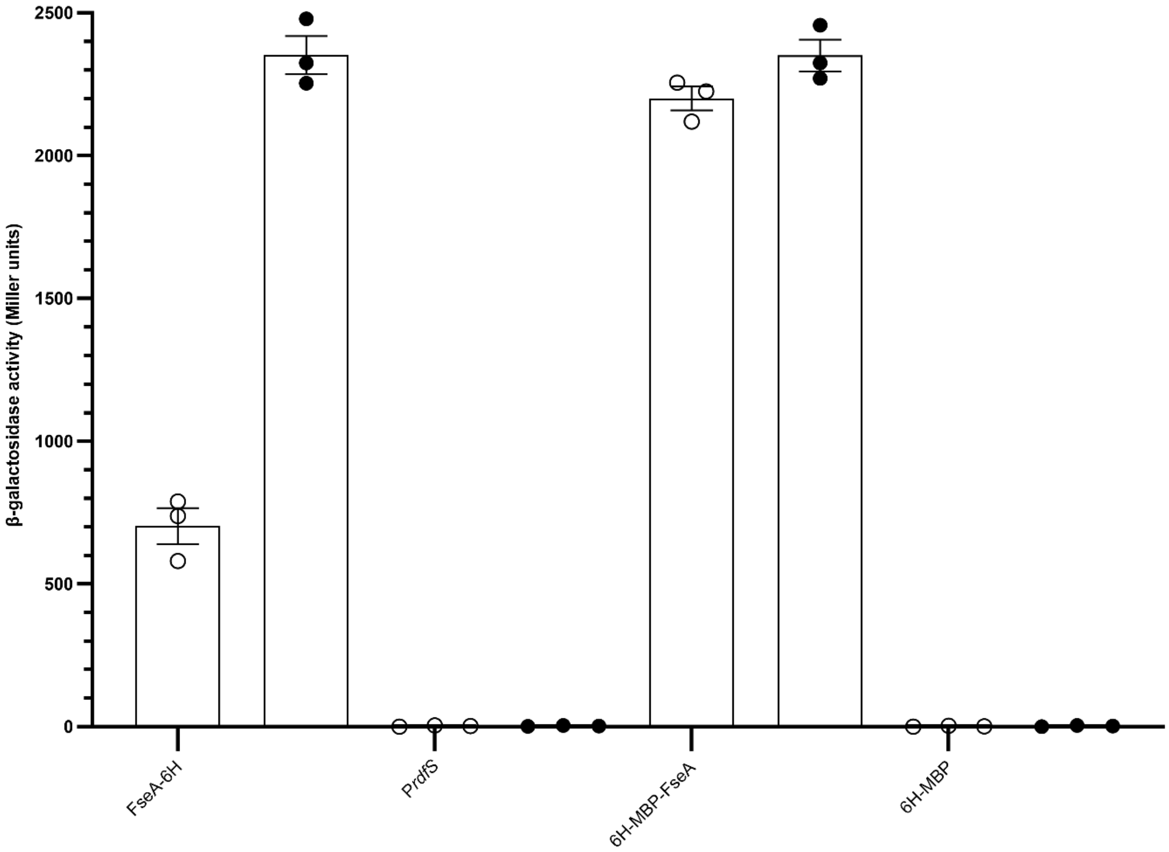

Figure S3. 6H-MBP-FseA activates transcription from P*rdfS.* P*rdfS* activation is represented by columns that show the mean β-galactosidase activity from three biological replicate assays, the individual values for which are denoted by circles (each an average of two technical replicates). Error bars show the SEM. Labels on the x-axis span two groups (uninduced, open circles; IPTG-induced *lac* promoter expression, filled circles).

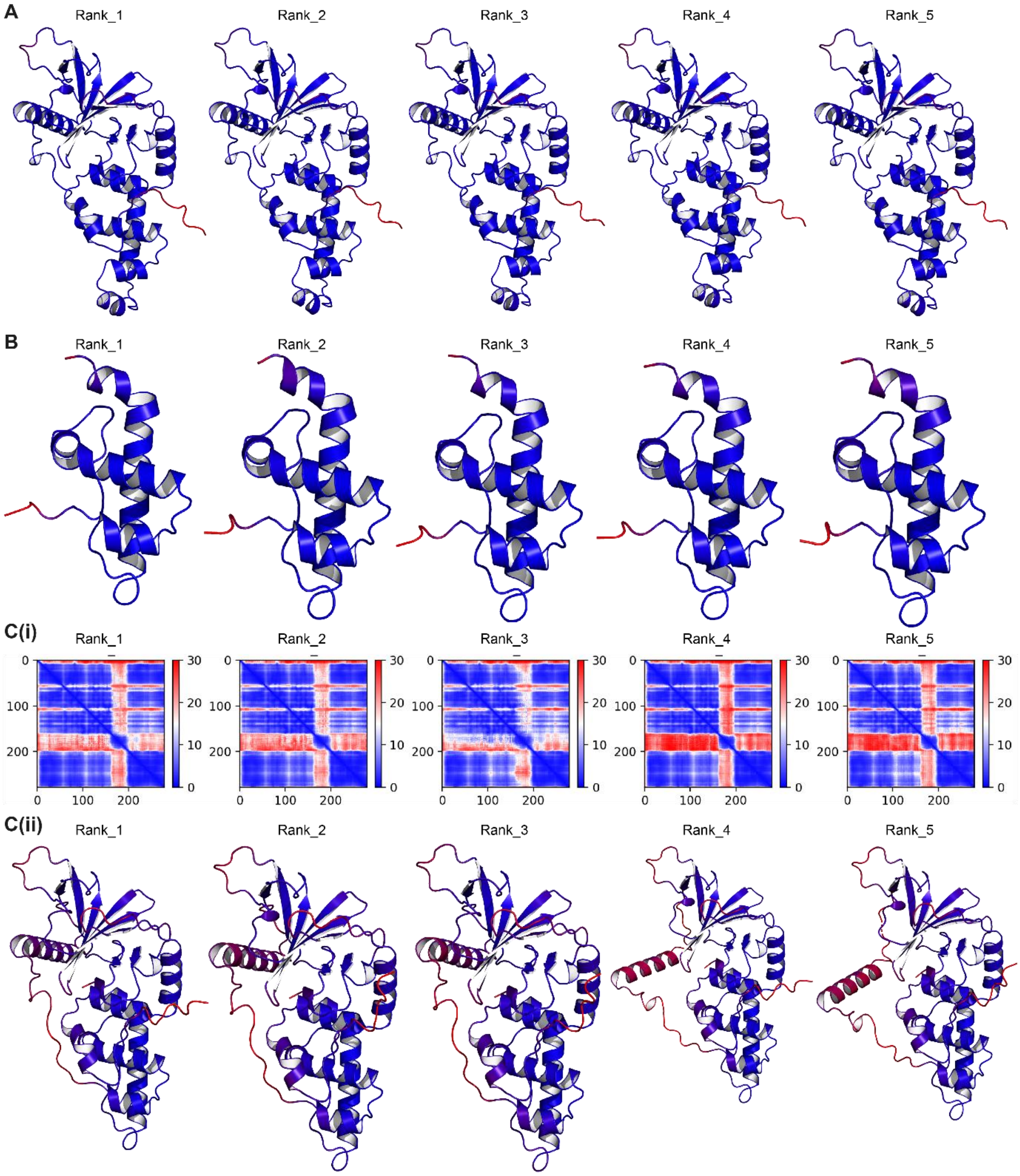

Figure S4. AF2 generated protein structure models are highly confident. Top five ranked models of (**A**) FseA, (**B**) QseM, and (**C(ii)**) the FseA-QseM fusion. In each model, amino acids are coloured by the confidence in their positions' using the calculated probability local deviation distance test value (blue, 1; red, <0.5). (**C(i)**) shows the predicted aligned error of the FseA-QseM fusion model.

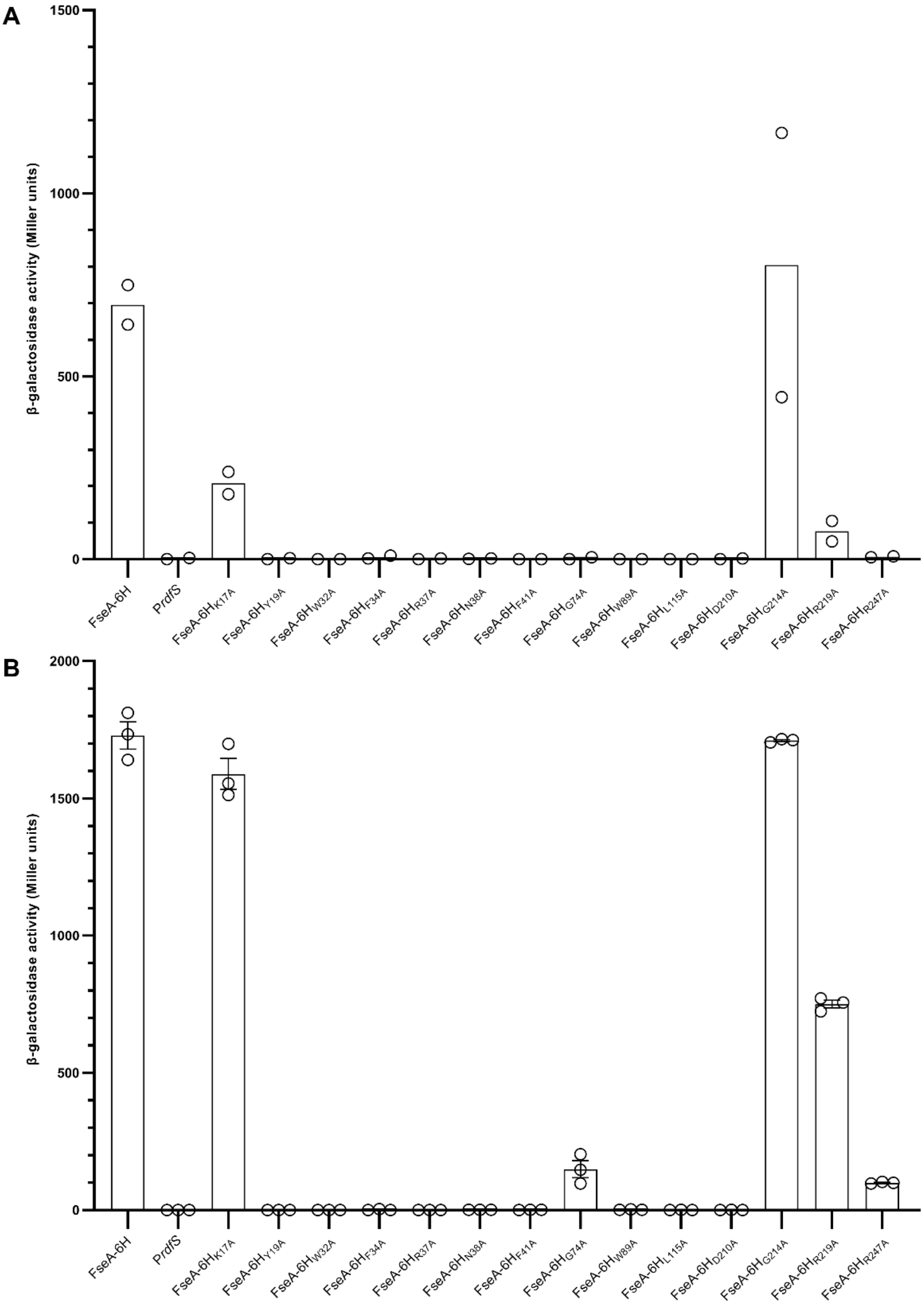

Figure S5. Mutation of FseA residues in the α2 helix typically abolishes P*rdfS* activation. P*rdfS* activation is represented by columns that show the mean β-galactosidase activity from two biological replicate (**A**) or three biological replicate assays (**B**), the individual values for which are denoted by circles (each an average of two technical replicates). Error bars show SEM. Samples in (**A**) were not induced, while samples in (**B**) were IPTG-induced for increased *lac* promoter expression. Values were normalised by the proportional difference of a single representative experiment's positive control and the respective experiment's positive control (performed independently in (**A**) and (**B**)).

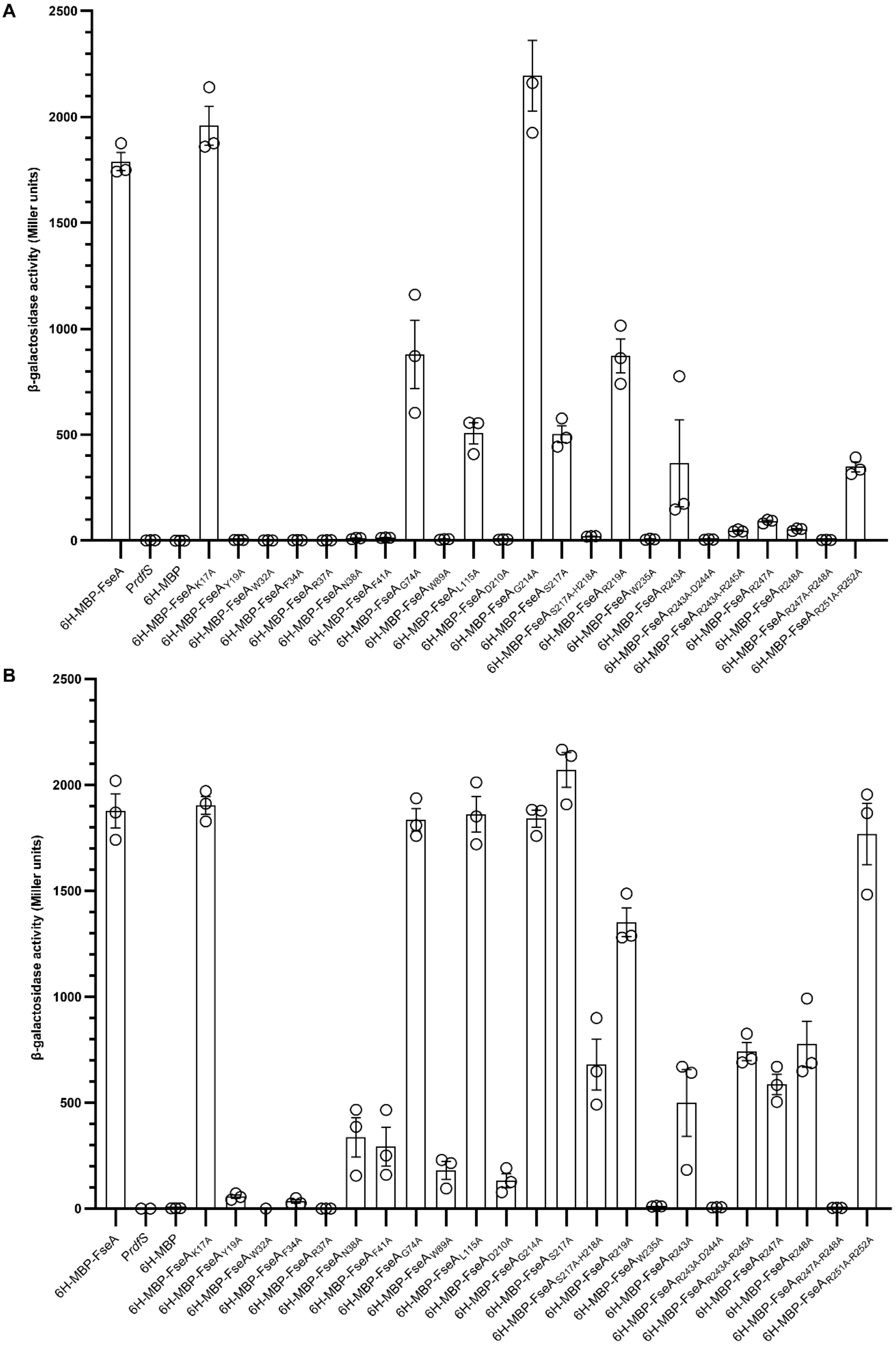

Figure S6. FseA residues key to P*rdfS* activation of MBP-FseA. P*rdfS* activation is represented by columns that show the mean β-galactosidase activity from three biological replicate assays, the individual values for which are denoted by circles (each an average of two technical replicates). Error bars show SEM. Samples in (**A**) were not induced, while samples in (**B**) were IPTG-induced for increased *lac* promoter expression. Values were normalised by the proportional difference of a single representative experiment's positive control and the respective experiment's positive control (performed independently in (**A**) and (**B**)).

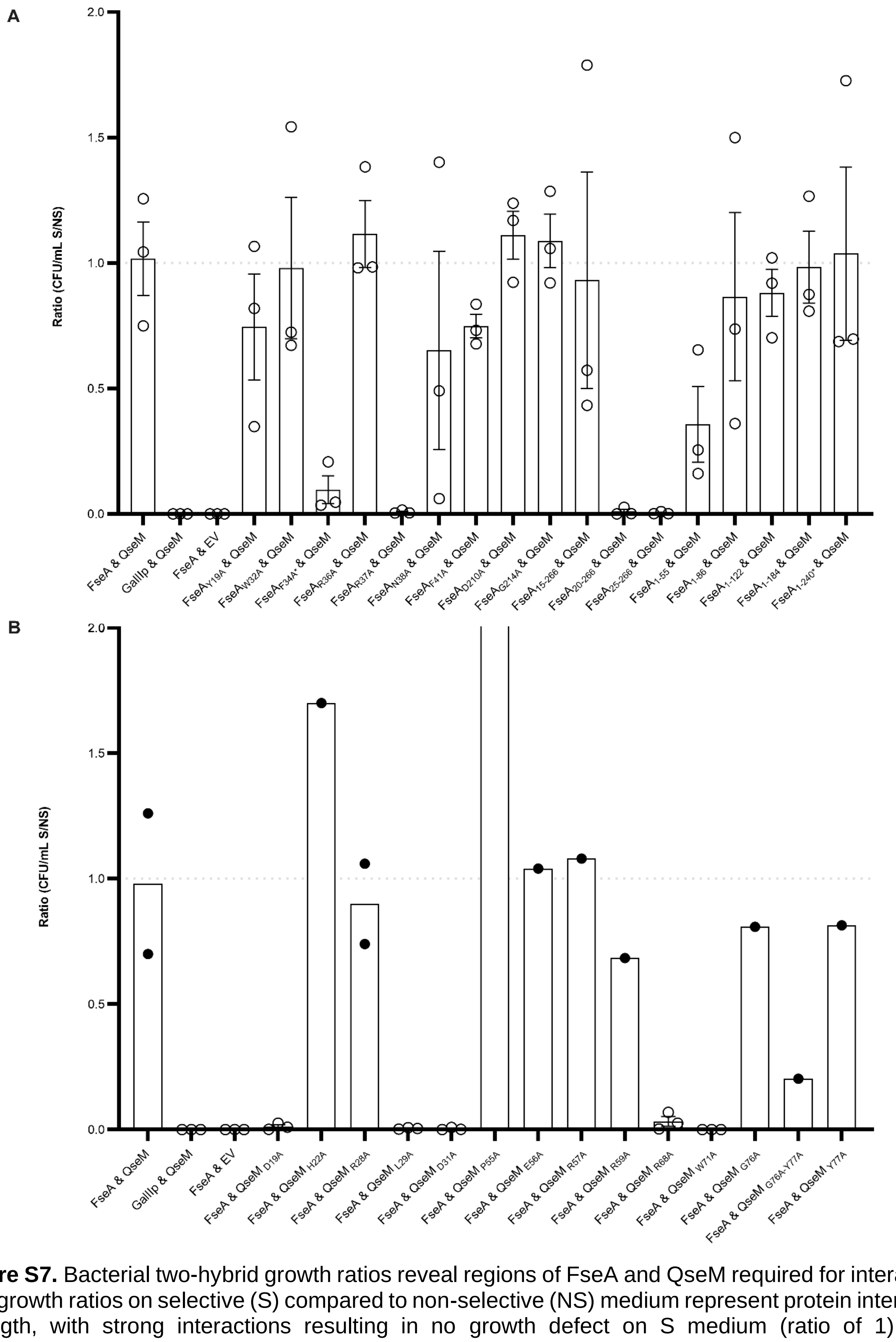

Figure S7. Bacterial two-hybrid growth ratios reveal regions of FseA and QseM required for interaction. Cell-growth ratios on selective (S) compared to non-selective (NS) medium represent protein interaction strength, with strong interactions resulting in no growth defect on S medium (ratio of 1). RNA polymerase α-subunit-fused GalIIp or FseA was expressed from the pTRG plasmid, while the λ-cI-QseM fusion was expressed from the pBT plasmid. EV represents pBT with λ-cI alone. Error bars show SEM. Biological replicate values (average of three technical replicates) are shown with circles. Open circles denote samples that were assayed in biological triplicate, while filled circles denote samples assayed in one or two biological replicates. (**A**) Ratios with QseM and mutated or truncated FseA. (**B**) Ratios with FseA and mutated QseM. The ratio of FseA & $QseM_{P55A}$ is 2.9. Biological replicate values were normalised separately in (**A**) and (**B**) by the proportional difference of a single representative experiment's FseA and QseM cell-growth ratio and the respective experiment's FseA and QseM ratio.

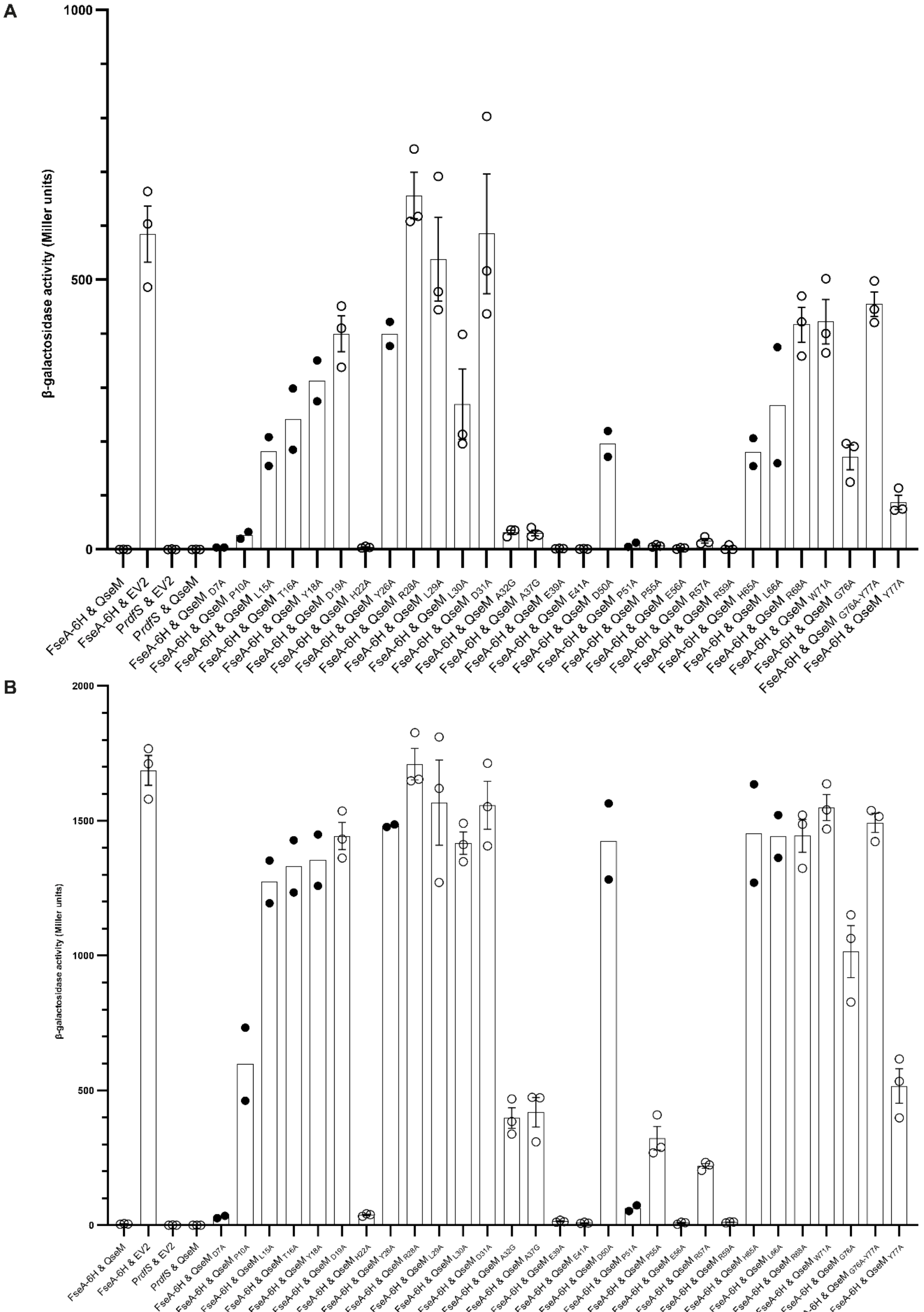

Figure S8. Alanine scanning mutagenesis of QseM reveals residues required for antiactivation of FseA-dependent P*rdfS* activation. Mean P*rdfS* activation is represented by columns, with circles denoting values from biological replicate assays (each an average of two technical replicates). Filled circles, biological duplicate; open, triplicate. Error bars show SEM for tests performed in biological triplicate. EV2 represents the empty pPR3G plasmid. Samples in (**A**) were not induced, while samples in (**B**) were IPTG-induced for increased *lac* promoter expression. Values were normalised by the proportional difference of a single representative experiment's positive control and the respective experiment's positive control (performed independently in (**A**) and (**B**)).

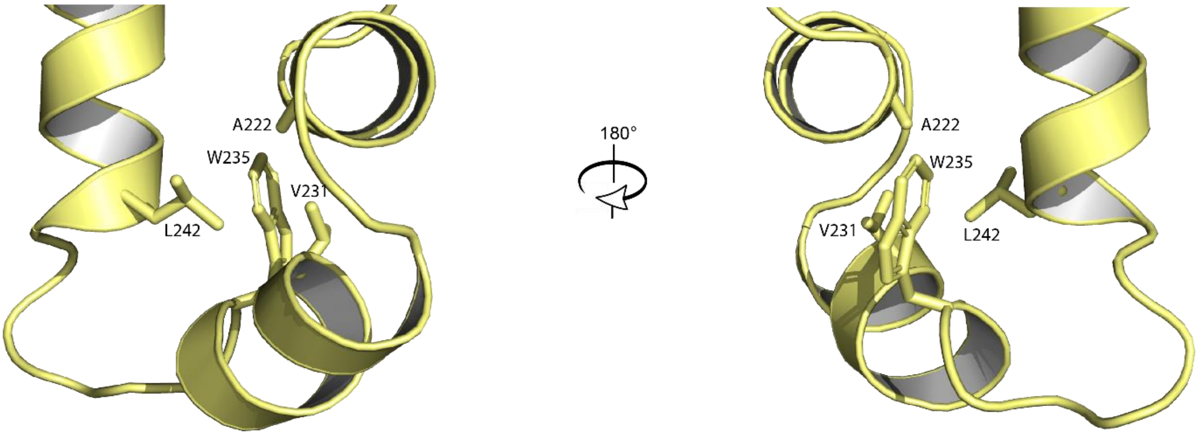

Figure S9. Trp235 is likely a key structural residue of the DUF2285 HTH that is required the domain to function correctly. Displayed is Trp235, Ala222, Val231, and Leu242 that together form a hydrophobic pocket between H2, H2b, and H3 of the DUF2285 HTH.

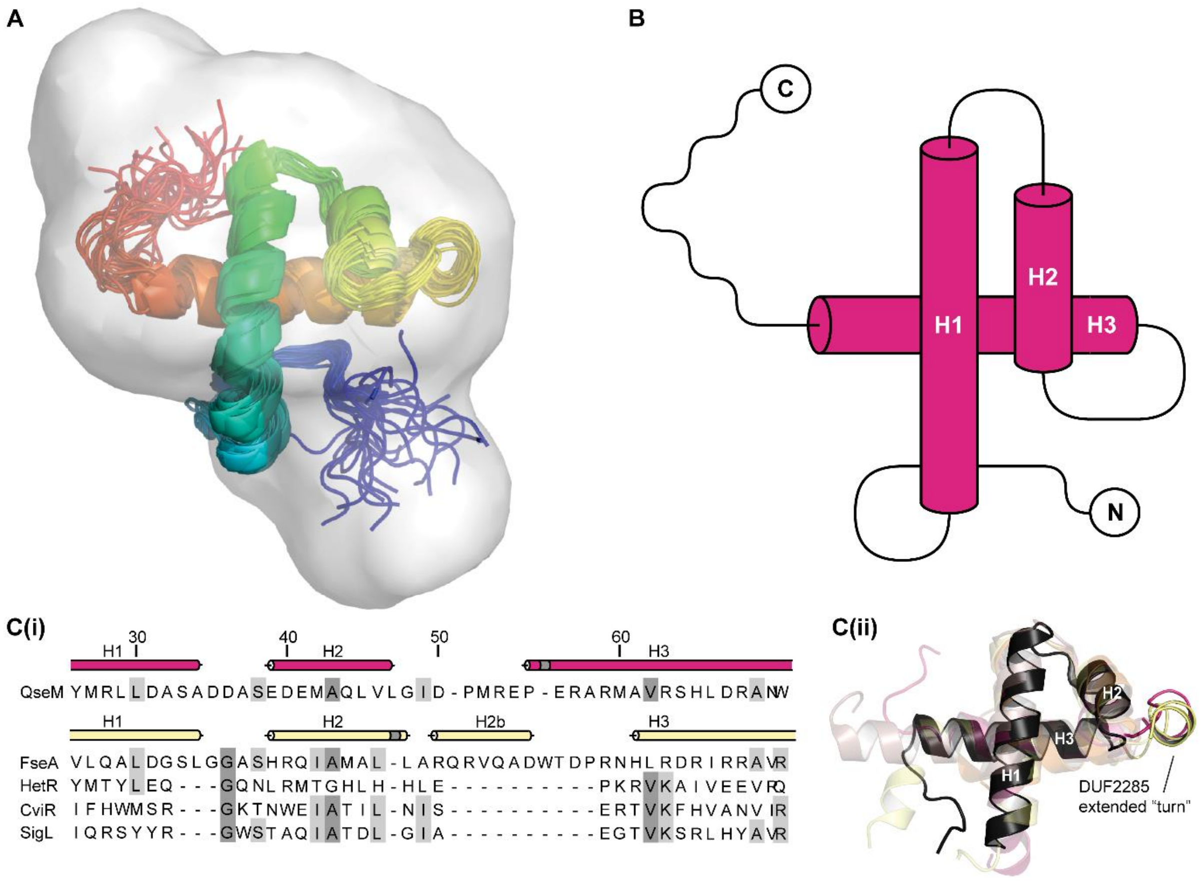

Figure S10. The QseM DUF2285 domain and comparison of the DUF2285 domain with DALI-identified HTH domains. (**A**) NMR structure ensemble (20 models) of QseM, coloured from N- (blue) to C-terminus (red), overlaid onto the envelope of an *ab initio* model derived from SAXS. (**B**) Topology diagram of QseM denoting the naming convention of its α-helices. (**C**) (**i**) Sequence alignment and secondary structure diagram of QseM, FseA and DALI-identified HTH domain-containing proteins (HetR, PDB 4izz; CviR, PDB 3qp6; and SigL, PDB 3hug), illustrating the extended turn present in QseM (pink) and FseA (yellow). Grey shading represents amino-acid similarity. Gaps in the sequence alignment are shown as grey sections in the secondary structure cartoon. (**ii**) Structural alignment of QseM (pink), FseA (yellow), HetR (black), CviR, and SigL. The canonical HTH H1/2/3 helices and the extended turn of the DUF2285 domain are denoted.

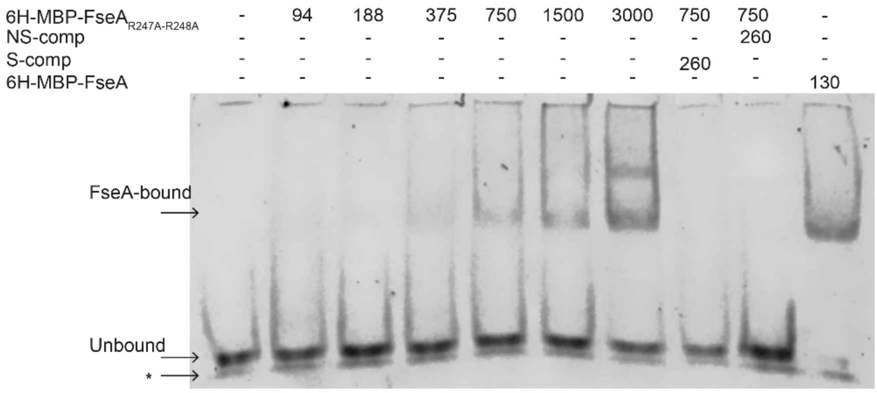

Figure S11. Reduced DNA-binding activity of $FseA_{R247A-R248A}$. EMSA performed with fluorescent FseA-box DNA and purified 6H-MBP-$FseA_{R247A-R248A}$ (94-3000 nM). Binding by wild-type 6H-MBP-FseA at 130 nM is also shown. NS- and S-comp denote added unlabelled non-specific and specific competitor DNA, respectively. The asterisk denotes fluorescent co-purified FseA-box DNA that remains equally unbound at all protein concentrations.

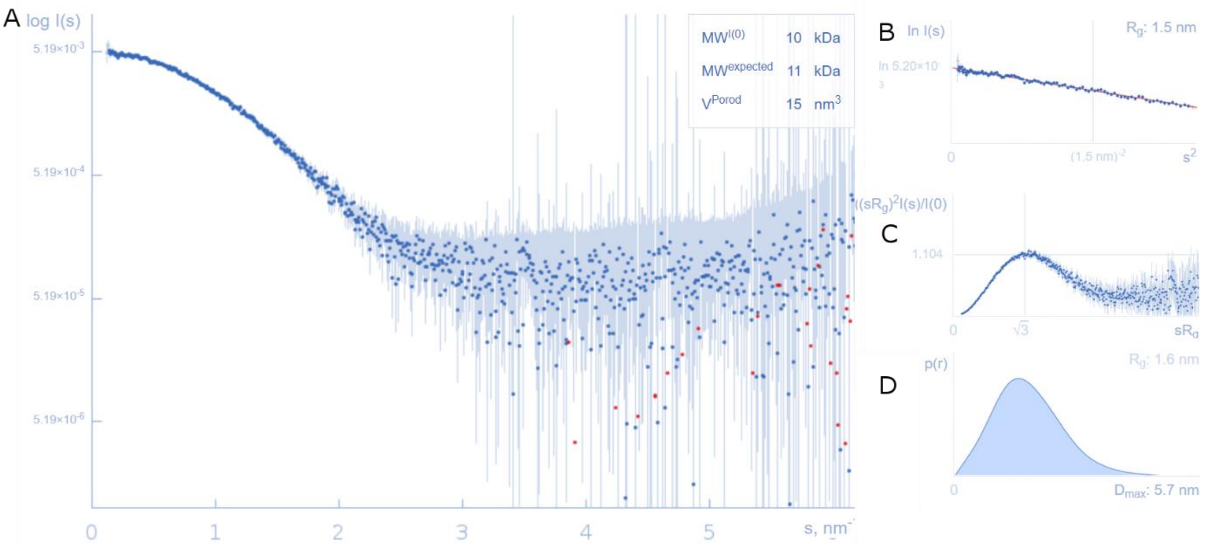

Figure S12. 6H-QseM SEC-SY-SAXS plots. SAXS data collected of 6H-QseM via SEC-SY-SAXS method and submitted to SASBDB under accession code SASDNM8. (**A**) Collected scattering intensities over resolution. (**B**) Guinier plot showing a monodisperse structure with a radius of gyration (Rg) of 1.5 nm. (**C**) Kratky plot showing that the protein is folded. (**D**) Pair distance distribution function revealing maximum diameter of the protein (Dmax) at 5.7 nm.

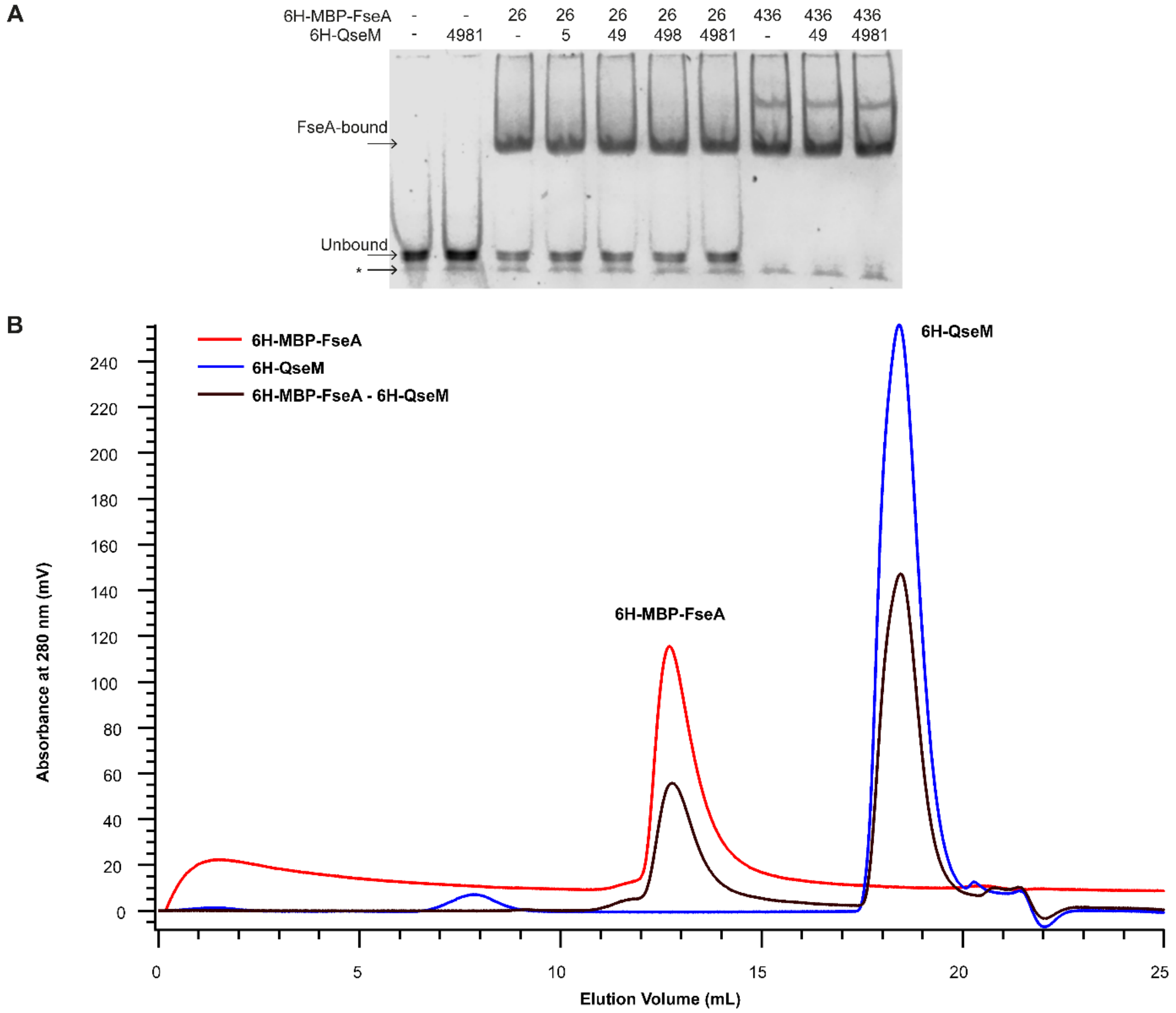

Figure S13. 6H-QseM does not bind P*rdfS* DNA or affect 6H-MBP-FseA DNA binding activity, and it did not bind 6H-MBP-FseA dimers in SEC-MALS experiments. (**A**) EMSA performed with fluorescent-labelled FseA-box DNA and 6H-MBP-FseA dimer concentrations of 26 or 436 nM and 6H-QseM concentrations of 5-4981 nM. The asterisk denotes fluorescent co-purified FseA-box DNA that remains equally unbound at all 6H-MBP-FseA concentrations. (**B**) Chromatography traces (UV280 absorbance) from SEC-MALS experiments of 6H-MBP-FseA dimers (red), 6H-QseM (blue), and 6H-MBP-FseA combined with 6H-QseM (black).

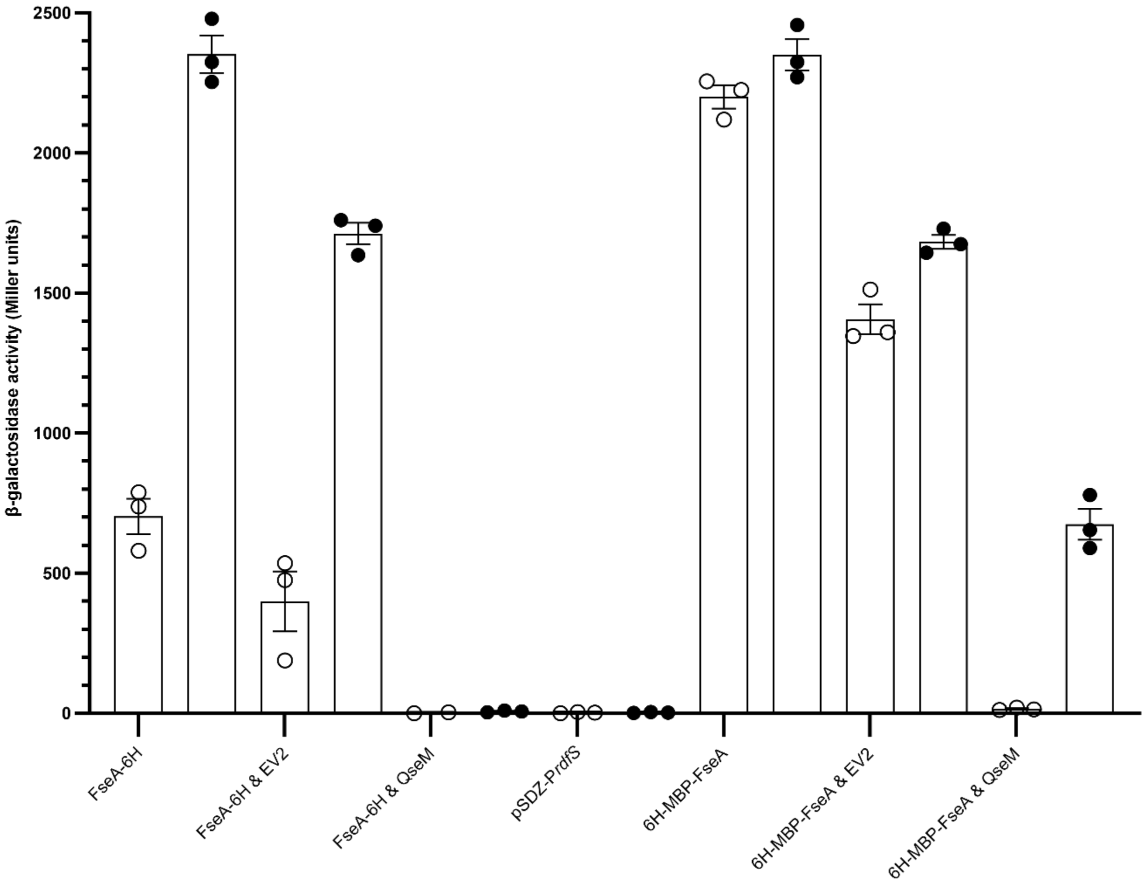

Figure S14. QseM antiactivates P*rdfS* activation by FseA-6H and 6H-MBP-FseA. P*rdfS* activation is represented by columns that show the mean β-galactosidase activity from three biological replicate assays, the individual values for which are denoted by circles (each an average of two technical replicates). Error bars show SEM. Samples with IPTG-induced *lac* promoter expression of the inserted gene are denoted with filled circles and uninduced expression by open circles. Labels on the x-axis span two groups (uninduced/induced). EV2 represents the empty pPR3G plasmid.

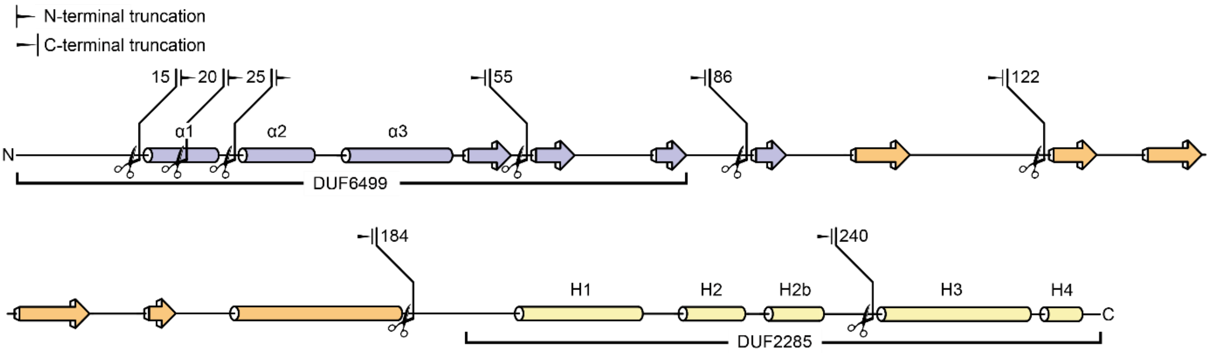

Figure S15. Sites used to produce truncations of FseA for identifying regions that interact with QseM in bacterial two-hybrid experiments. Secondary structure of the AF2 FseA structure coloured by domain as in Figure 3B. Scissor symbols and accompanying lines indicate the positions (denoted) at which the FseA truncations were made and assayed by bacterial two-hybrid assays in Figure S7A.

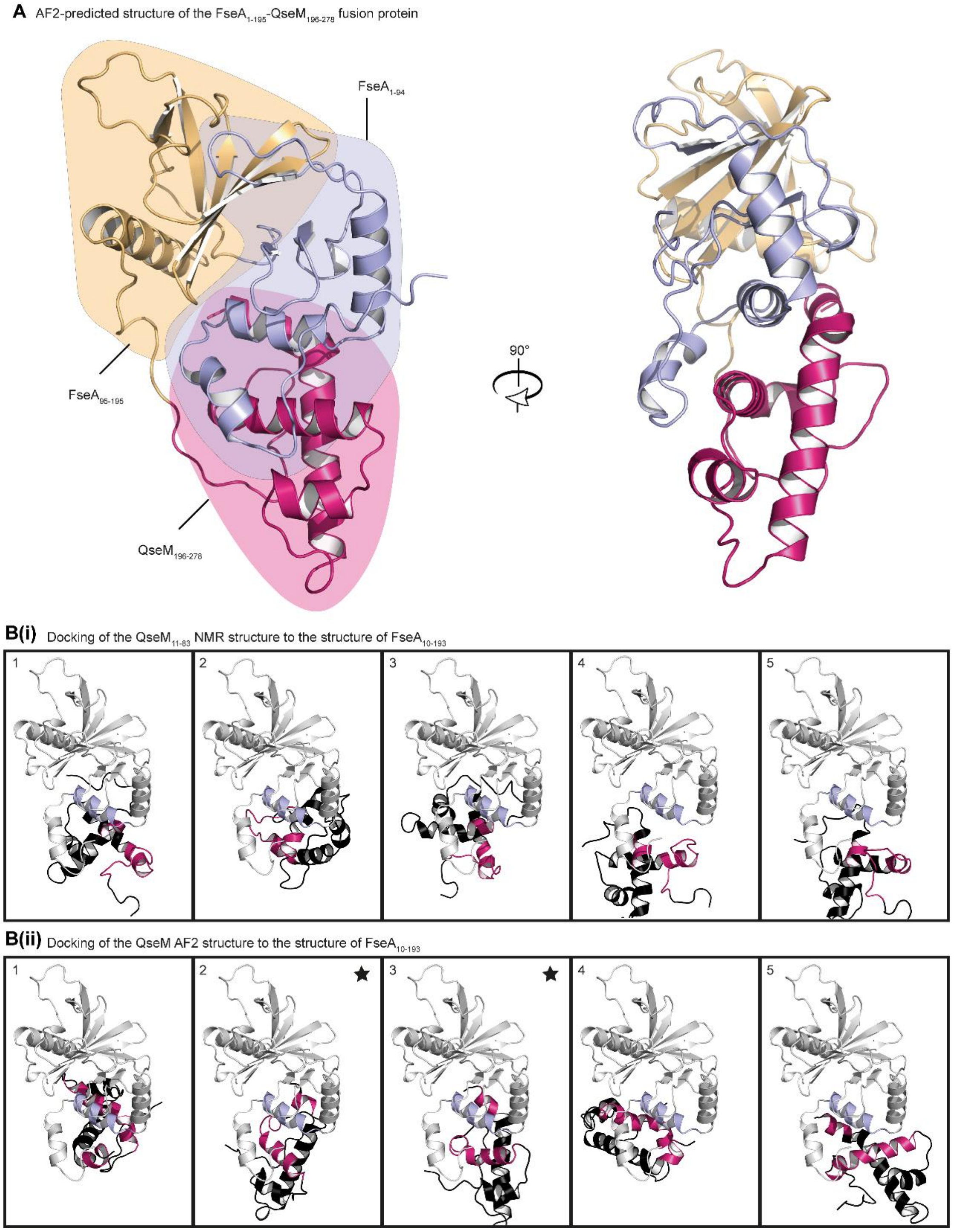

Figure S16. Computational prediction of the FseA-QseM interaction supports DUF6499-binding of QseM and presence of H4 of QseM is required for this interaction to occur in docking simulations. (**A**) AF2 predicted structure of the $FseA_{1\text{-}195}$-$QseM_{196\text{-}278}$ fusion protein. The FseA DUF6499 domain, blue; middle domain, orange; and QseM, pink. The five top-ranked models generated by AF2 are shown in Figure S4C. (**B**) Top 5 ClusPro docking predictions of the interaction of $FseA_{10\text{-}193}$ and (**i**) the $QseM_{11\text{-}83}$ NMR structure and (**ii**) the AF2-predicted QseM structure. Black stars denote predictions that approximate FseA-QseM fusion interaction.

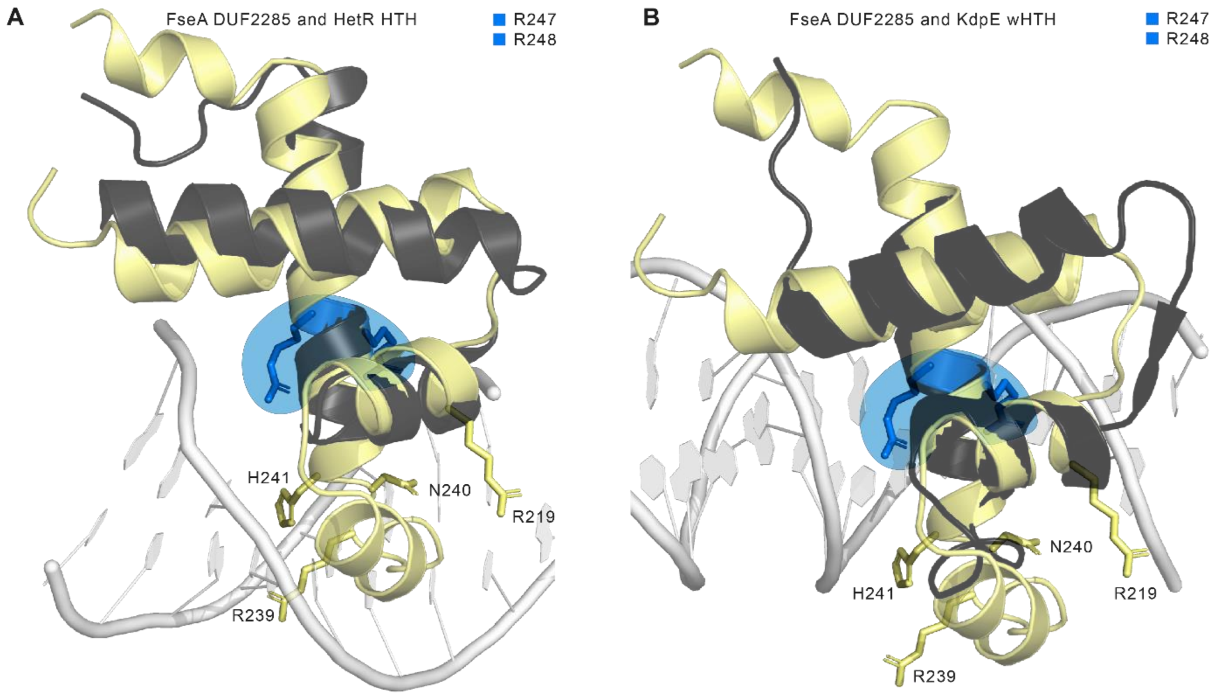

Figure S17. Possible modes of DNA binding of the FseA DUF2285 domain. Alignment of the FseA DUF2285 domain (yellow) to (**A**) HetR (PBD 4IZZ) and (**B**) KdpE (PDB 4KNY). Co-crystalized DNA is shown in silver. Side chains of residues in the extended turn of the FseA DUF2285 that are close to the DNA of the aligned structure are shown, and Arg247 and Arg248 that are required for strong DNA binding are shown in blue.

Table S1. Bacterial strains used in this study

Strain	Characteristics	Source
E. coli		
BL21(DE3)pLysS	*fhuA2 [lon] ompT gal (λ DE3) [dcm] ∆hsdS λ DE3 = λ sBamHIo ∆EcoRI-B int::(lacI::PlacUV5::T7 gene1) i21 ∆nin5*, pLysS, Cm^r	1
EPI300	F^- *mcrA Δ(mrr-hsdRMS-mcrBC) φ80dlacZΔM15 ΔlacX74 recA1 endA1 araD139 Δ(ara, leu)7697 galU galK* λ^- *rpsL nupG trfA tonA dhrf,* Str^R	Epicentre® Biotechnologies
NiCo21(DE3)	*can::CBD fhuA2 [lon] ompT gal (λ DE3) [dcm] arnA::CBD slyD::CBD glmS6Ala ∆hsdS λ DE3 = λ sBamHIo ∆EcoRI-B int::(lacI::PlacUV5::T7 gene1) i21 ∆nin5*	2
S17-1	*pro, res*$^-$ *hsdR17 (rK*$^-$ *mK*$^+$*) recA*$^-$ *RP4-2-Tc::Mu-Km::Tn7*, Tp^r	3
ST18	S17 Δ*hemA*	4
XL1-Blue MRF' Kan	∆(*mcrA*)*183* ∆(*mcrCB-hsdSMR-mrr*)*173 endA1 supE44 thi-1 recA1 gyrA96 relA1 lac* [F´ *proAB lacIqZ∆M15 Tn5* Kan^R]	Agilent Technologies Cat. 200248
Bacteriomatch II Two-Hybrid System Report Strain	∆(*mcrA*)*183* ∆(*mcrCB-hsdSMR-mrr*)*173 endA1 hisB supE44 thi-1 recA1 gyrA96 relA1 lac* [F´ *laqIq HIS3 aadA* Kan^R]	Agilent Technologies Cat. 200192
M. japonicum		
R7A	Field reisolate of ICMP 3153; wild-type symbiotic strain	5
R7ANS	Non-symbiotic derivative of R7A, lacks ICE*Ml*Sym^{R7A}	6

Table S2. Plasmids used in this study

plasmid	Description	Source
pBT	Bacteriomatch II Two-hybrid bait cloning vector. P15A origin, Cm^R, *lac-UV5* promoter with the λcI ORF fused	Agilent Technologies
pBT-*LGF2*	Bacteriomatch II Two-hybrid positive control plasmid	Agilent Technologies
pBT-*qseM*	pBT containing *6H-qseM* fused to the *λcI* ORF	7
pBT-$qseM_{D19A}$	pBT-*qseM* with *qseM::D19A*	This study
pBT-$qseM_{H22A}$	pBT-*qseM* with *qseM::H22A*	This study
pBT-$qseM_{R28A}$	pBT-*qseM* with *qseM::R28A*	This study
pBT-$qseM_{L29A}$	pBT-*qseM* with *qseM::L29A*	This study
pBT-$qseM_{D31A}$	pBT-*qseM* with *qseM::D31A*	This study
pBT-$qseM_{P55A}$	pBT-*qseM* with *qseM::P55A*	This study
pBT-$qseM_{E56A}$	pBT-*qseM* with *qseM::E56A*	This study
pBT-$qseM_{R57A}$	pBT-*qseM* with *qseM::R57A*	This study
pBT-$qseM_{R59A}$	pBT-*qseM* with *qseM::R59A*	This study
pBT-$qseM_{R68A}$	pBT-*qseM* with *qseM::R68A*	This study
pBT-$qseM_{W71A}$	pBT-*qseM* with *qseM::W71A*	This study
pBT-$qseM_{G76A}$	pBT-*qseM* with *qseM::G76A*	This study
pBT-$qseM_{G76A\text{-}Y77A}$	pBT-*qseM* with *qseM::GY76-77AA*	This study
pBT-$qseM_{Y77A}$	pBT-*qseM* with *qseM::Y77A*	This study
pPROBE-GT	Plasmid designed to probe promoter activity. P15A origin, Gm^R, promoterless *gfp*	8
pPR3G	pPROBE-GT containing a 336-bp region of the *nptII* promoter amplified from pFAJ1708 and cloned upstream of *gfp*	9
pPR3G-*qseM*	pPR3G containing 6His-*qseM* fused to the *nptII* promoter	This study
pPR3G-$qseM_{D7A}$	pPR3G-*qseM* with *qseM::D7A*	This study
pPR3G-$qseM_{P10A}$	pPR3G-*qseM* with *qseM::P10A*	This study
pPR3G-$qseM_{L15A}$	pPR3G-*qseM* with *qseM::L15A*	This study
pPR3G-$qseM_{T16A}$	pPR3G-*qseM* with *qseM::T16A*	This study
pPR3G-$qseM_{Y18A}$	pPR3G-*qseM* with *qseM::Y18A*	This study
pPR3G-$qseM_{D19A}$	pPR3G-*qseM* with *qseM::D19A*	This study
pPR3G-$qseM_{H22A}$	pPR3G-*qseM* with *qseM::H22A*	This study
pPR3G-$qseM_{Y26A}$	pPR3G-*qseM* with *qseM::Y26A*	This study
pPR3G-$qseM_{R28Q}$	pPR3G-*qseM* with *qseM::R28Q*	This study
pPR3G-$qseM_{L29A}$	pPR3G-*qseM* with *qseM::L29A*	This study

pPR3G-*qseM*$_{L30A}$	pPR3G-*qseM* with *qseM::L30A*	This study
pPR3G-*qseM*$_{D31A}$	pPR3G-*qseM* with *qseM::D31A*	This study
pPR3G-*qseM*$_{A32G}$	pPR3G-*qseM* with *qseM::A32G*	This study
pPR3G-*qseM*$_{A37G}$	pPR3G-*qseM* with *qseM::A37G*	This study
pPR3G-*qseM*$_{E39A}$	pPR3G-*qseM* with *qseM::E39A*	This study
pPR3G-*qseM*$_{E41A}$	pPR3G-*qseM* with *qseM::E41A*	This study
pPR3G-*qseM*$_{P51A}$	pPR3G-*qseM* with *qseM::P51A*	This study
pPR3G-*qseM*$_{E56A}$	pPR3G-*qseM* with *qseM::E56A*	This study
pPR3G-*qseM*$_{R57A}$	pPR3G-*qseM* with *qseM::R57A*	This study
pPR3G-*qseM*$_{R59A}$	pPR3G-*qseM* with *qseM::R59A*	This study
pPR3G-*qseM*$_{H65A}$	pPR3G-*qseM* with *qseM::H65A*	This study
pPR3G-*qseM*$_{L66A}$	pPR3G-*qseM* with *qseM::L66A*	This study
pPR3G-*qseM*$_{R68A}$	pPR3G-*qseM* with *qseM::R68A*	This study
pPR3G-*qseM*$_{W71A}$	pPR3G-*qseM* with *qseM::W71A*	This study
pPR3G-*qseM*$_{G76A}$	pPR3G-*qseM* with *qseM::G76A*	This study
pPR3G-*qseM*$_{Y77A}$	pPR3G-*qseM* with *qseM::Y77A*	This study
pQE80oriT	pQE-80L containing the RK2 origin of transfer cloned as an NdeI fragment, amplified from plasmid pFAJ1700	10
pQe80-*6H-qseM*	pQe80oriT carrying *qseM* fused downstream of the *6H*	This study
pSDZ	Plasmid designed to test coupled promoter-activator activity. pFAJ1700 (RK2-derived) origin, Tc^R, Ap^R, promoterless *lacZ*, *lac* promoter, linker-*gfp*, *lacI*	7
pSDZ-P*rdfS*	pSDZ containing the *rdfS* promoter region (211-bp fragment) fused to *lacZ*	7
pSDZ-P*rdfS-fseA-6H*	pSDZ-P*rdfS* containing frameshifted *fseA-6H* fused to the *lac* promoter	7
pSDZ-P*rdfS*$_{Tr38}$	pSDZ-P*rdfS* with P*rdfS* truncated at 38-bp upstream of the P*rdfS* start site	This study
pSDZ-P*rdfS*$_{Tr38}$-*fseA-6H*	pSDZ-P*rdfS*$_{Tr38}$ containing frameshifted *fseA-6H* fused to the *lac* promoter	This study
pSDZ-P*rdfS*$_{Tr45}$	pSDZ-P*rdfS* with P*rdfS* truncated at 45-bp upstream of the P*rdfS* start site	This study
pSDZ-P*rdfS*$_{Tr45}$-*fseA-6H*	pSDZ-P*rdfS*$_{Tr45}$ containing frameshifted *fseA-6H* fused to the *lac* promoter	This study
pSDZ-P*rdfS*$_{Tr61}$	pSDZ-P*rdfS* with P*rdfS* truncated at 61-bp upstream of the P*rdfS* start site	This study
pSDZ-P*rdfS*$_{Tr61}$-*fseA-6H*	pSDZ-P*rdfS*$_{Tr61}$ containing frameshifted *fseA-6H* fused to the *lac* promoter	This study
pSDZ-P*rdfS*$_{Tr67}$	pSDZ-P*rdfS* with P*rdfS* truncated at 67-bp upstream of the P*rdfS* start site	This study

pSDZ-P*rdfS*$_{Tr67}$-*fseA-6H*	pSDZ-P*rdfS*$_{Tr67}$ containing frameshifted *fseA-6H* fused to the *lac* promoter	This study
pSDZ-P*rdfS*$_{Tr73}$	pSDZ-P*rdfS* with P*rdfS* truncated at 73-bp upstream of the P*rdfS* start site	This study
pSDZ-P*rdfS*$_{Tr73}$-*fseA-6H*	pSDZ-P*rdfS*$_{Tr73}$ containing frameshifted *fseA-6H* fused to the *lac* promoter	This study
pSDZ-P*rdfS*$_{C41A}$-*fseA-6H*	pSDZ-P*rdfS-fseA-6H* with P*rdfS* substitution of C/A 41-bp upstream of the P*rdfS* start site	This study
pSDZ-P*rdfS*$_{C43G}$-*fseA-6H*	pSDZ-P*rdfS-fseA-6H* with P*rdfS* substitution of C/G 43-bp upstream of the P*rdfS* start site	This study
pSDZ-P*rdfS*$_{C46T}$-*fseA-6H*	pSDZ-P*rdfS-fseA-6H* with P*rdfS* substitution of C/T 46-bp upstream of the P*rdfS* start site	This study
pSDZ-P*rdfS*$_{A49T}$-*fseA-6H*	pSDZ-P*rdfS-fseA-6H* with P*rdfS* substitution of A/T 49-bp upstream of the P*rdfS* start site	This study
pSDZ-P*rdfS*$_{C52T}$-*fseA-6H*	pSDZ-P*rdfS-fseA-6H* with P*rdfS* substitution of C/T 52-bp upstream of the P*rdfS* start site	This study
pSDZ-P*rdfS*$_{G64C}$-*fseA-6H*	pSDZ-P*rdfS-fseA-6H* with P*rdfS* substitution of G/C 64-bp upstream of the P*rdfS* start site	This study
pSDZ-P*rdfS*$_{G66T}$-*fseA-6H*	pSDZ-P*rdfS-fseA-6H* with P*rdfS* substitution of G/T 66-bp upstream of the P*rdfS* start site	This study
pSDZ-P*rdfS*$_{Rev1}$-*fseA-6H*	pSDZ-P*rdfS-fseA-6H* with P*rdfS* substitution of 5′-TTCGCC-3′ to 5′-GGCGAA-3′ at base-pair positions 40-45 upstream of the P*rdfS* start site	This study
pSDZ-P*rdfS*$_{Rev2}$-*fseA-6H*	pSDZ-P*rdfS-fseA-6H* with P*rdfS* substitution of 5′-GGCGAA-3′ to 5′-TTCGCC-3′ at base-pair positions 62-67 upstream of the P*rdfS* start site	This study
pSDZ-P*rdfS-fseA-6H*$_{K17A}$	pSDZ-P*rdfS-fseA-6H* with *fseA::K17A*	This study
pSDZ-P*rdfS-fseA-6H*$_{Y19A}$	pSDZ-P*rdfS-fseA-6H* with *fseA::Y19A*	This study
pSDZ-P*rdfS-fseA-6H*$_{W32A}$	pSDZ-P*rdfS-fseA-6H* with *fseA::W32A*	This study
pSDZ-P*rdfS-fseA-6H*$_{F34A}$	pSDZ-P*rdfS-fseA-6H* with *fseA::F34A*	This study
pSDZ-P*rdfS-fseA-6H*$_{R37A}$	pSDZ-P*rdfS-fseA-6H* with *fseA::R37A*	This study
pSDZ-P*rdfS-fseA-6H*$_{N38A}$	pSDZ-P*rdfS-fseA-6H* with *fseA::N38A*	This study
pSDZ-P*rdfS-fseA-6H*$_{F41A}$	pSDZ-P*rdfS-fseA-6H* with *fseA::F41A*	This study

pSDZ-P*rdfS-fseA-6H*$_{G74A}$	pSDZ-P*rdfS-fseA-6H* with *fseA::G74A*	This study
pSDZ-P*rdfS-fseA-6H*$_{W89A}$	pSDZ-P*rdfS-fseA-6H* with *fseA::W89A*	This study
pSDZ-P*rdfS-fseA-6H*$_{L115A}$	pSDZ-P*rdfS-fseA-6H* with *fseA::L115A*	This study
pSDZ-P*rdfS-fseA-6H*$_{D210A}$	pSDZ-P*rdfS-fseA-6H* with *fseA::D210A*	This study
pSDZ-P*rdfS-fseA-6H*$_{G214A}$	pSDZ-P*rdfS-fseA-6H* with *fseA::G214A*	This study
pSDZ-P*rdfS-fseA-6H*$_{R219A}$	pSDZ-P*rdfS-fseA-6H* with *fseA::R219A*	This study
pSDZ-P*rdfS-fseA-6H*$_{R247A}$	pSDZ-P*rdfS-fseA-6H* with *fseA::R247A*	This study
pSDZ-P*rdfS-6H-MBP*	pSDZ-P*rdfS* containing *6H-MBP*	This study
pSDZ-P*rdfS-6H-MBP-fseA*	pSDZ-P*rdfS* containing *6H-MBP-fseA*	This study
pSDZ-P*rdfS-6H-MBP-fseA*$_{K17A}$	pSDZ-P*rdfS-6H-MBP-fseA* with *fseA::K17A*	This study
pSDZ-P*rdfS-6H-MBP-fseA*$_{Y19A}$	pSDZ-P*rdfS-6H-MBP-fseA* with *fseA::Y19A*	This study
pSDZ-P*rdfS-6H-MBP-fseA*$_{W32A}$	pSDZ-P*rdfS-6H-MBP-fseA* with *fseA::W32A*	This study
pSDZ-P*rdfS-6H-MBP-fseA*$_{F34A}$	pSDZ-P*rdfS-6H-MBP-fseA* with *fseA::F34A*	This study
pSDZ-P*rdfS-6H-MBP-fseA*$_{R37A}$	pSDZ-P*rdfS-6H-MBP-fseA* with *fseA::R37A*	This study
pSDZ-P*rdfS-6H-MBP-fseA*$_{N38A}$	pSDZ-P*rdfS-6H-MBP-fseA* with *fseA::N38A*	This study
pSDZ-P*rdfS-6H-MBP-fseA*$_{F41A}$	pSDZ-P*rdfS-6H-MBP-fseA* with *fseA::F41A*	This study
pSDZ-P*rdfS-6H-MBP-fseA*$_{G74A}$	pSDZ-P*rdfS-6H-MBP-fseA* with *fseA::G74A*	This study
pSDZ-P*rdfS-6H-MBP-fseA*$_{W89A}$	pSDZ-P*rdfS-6H-MBP-fseA* with *fseA::W89A*	This study
pSDZ-P*rdfS-6H-MBP-fseA*$_{L115A}$	pSDZ-P*rdfS-6H-MBP-fseA* with *fseA::L115A*	This study
pSDZ-P*rdfS-6H-MBP-fseA*$_{D210A}$	pSDZ-P*rdfS-6H-MBP-fseA* with *fseA::D210A*	This study

pSDZ-P*rdfS-6H-MBP-fseA*$_{G214A}$	pSDZ-P*rdfS-6H-MBP-fseA* with *fseA::G214A*	This study
pSDZ-P*rdfS-6H-MBP-fseA*$_{S217A}$	pSDZ-P*rdfS-6H-MBP-fseA* with *fseA::S217A*	This study
pSDZ-P*rdfS-6H-MBP-fseA*$_{SH217\text{-}218AA}$	pSDZ-P*rdfS-6H-MBP-fseA* with *fseA::SH217-218AA*	This study
pSDZ-P*rdfS-6H-MBP-fseA*$_{R219A}$	pSDZ-P*rdfS-6H-MBP-fseA* with *fseA::R219A*	This study
pSDZ-P*rdfS-6H-MBP-fseA*$_{W235A}$	pSDZ-P*rdfS-6H-MBP-fseA* with *fseA::W235A*	This study
pSDZ-P*rdfS-6H-MBP-fseA*$_{R243A}$	pSDZ-P*rdfS-6H-MBP-fseA* with *fseA::R243A*	This study
pSDZ-P*rdfS-6H-MBP-fseA*$_{R243A\text{-}D244A}$	pSDZ-P*rdfS-6H-MBP-fseA* with *fseA::R243A-D244A*	This study
pSDZ-P*rdfS-6H-MBP-fseA*$_{R247A}$	pSDZ-P*rdfS-6H-MBP-fseA* with *fseA::R247A*	This study
pSDZ-P*rdfS-6H-MBP-fseA*$_{R248A}$	pSDZ-P*rdfS-6H-MBP-fseA* with *fseA::R248A*	This study
pSDZ-P*rdfS-6H-MBP-fseA*$_{R247A\text{-}R248A}$	pSDZ-P*rdfS-6H-MBP-fseA* with *fseA::R247A-R248A*	This study
pSDZ-P*rdfS-6H-MBP-fseA*$_{R251A\text{-}R252A}$	pSDZ-P*rdfS-6H-MBP-fseA* with *fseA::R251A-R252A*	This study
pTRG	Bacteriomatch II Two-hybrid target cloning vector. Ori^{ColE1}, Tc^{R}, *lpp*/*lac-UV5* promoter fused to RNA polymerase α-subunit ORF	Agilent Technologies
pTRG-*GalII*^{p}	pTRG containing a *galIIp* domain fused to the RNA polymerase α-subunit ORF	Agilent Technologies
pTRG-*fseA*	pTRG containing *fseA* fused to the RNA polymerase α-subunit ORF	7
pTRG-*fseA*$_{1\text{-}55}$	pTRG containing *fseA* C-terminally truncated (CTT) at 55 amino acids	This study
pTRG-*fseA*$_{1\text{-}86}$	pTRG containing *fseA* CTT at 86 amino acids	This study
pTRG-*fseA*$_{1\text{-}122}$	pTRG containing *fseA* CTT at 122 amino acids	This study
pTRG-*fseA*$_{1\text{-}184}$	pTRG containing *fseA* CTT at 184 amino acids	This study
pTRG-*fseA*$_{1\text{-}240*}$	pTRG containing *fseA* CTT at 240 amino acids; *fseA* harbours mutation R239L	This study
pTRG-*fseA*$_{15\text{-}266}$	pTRG containing *fseA* N-terminally truncated (NTT) at amino acid 15	This study
pTRG-*fseA*$_{20\text{-}266}$	pTRG containing *fseA* NTT at amino acid 20	This study
pTRG-*fseA*$_{25\text{-}266}$	pTRG containing *fseA* NTT at amino acid 25	This study

pTRG-*fseA*$_{Y19A}$	pTRG with *fseA::Y19A*	This study
pTRG-*fseA*$_{W32A}$	pTRG with *fseA::W32A*	This study
pTRG-*fseA*$_{F34A*}$	pTRG with *fseA::F34A-H172Y*	This study
pTRG-*fseA*$_{R36A}$	pTRG with *fseA::R36A*	This study
pTRG-*fseA*$_{R37A}$	pTRG with *fseA::R37A*	This study
pTRG-*fseA*$_{N38A}$	pTRG with *fseA::N38A*	This study
pTRG-*fseA*$_{F41A}$	pTRG with *fseA::F41A*	This study
pTRG-*fseA*$_{D210A}$	pTRG with *fseA::D210A*	This study
pTRG-*fseA*$_{G214A}$	pTRG with *fseA::G214A*	This study
pETM11	Protein expression vector. pBR322 origin, Km^R, Tc^R, T7 promoter with *6H-TEV* fused	EMBL Protein Expression and Purification
pETM11-*qseM*	pETM11 containing *qseM* fused to *6H-TEV*	This study
pETM41	Protein expression vector. pBR322 origin, Km^R, Tc^R, T7 promoter with *6H-TEV-MBP* fused	EMBL Protein Expression and Purification
pETM41-*fseA*	pETM41 containing codon optimised *fseA* fused to *6H-TEV-MBP*	This study
pETM41-*fseA*$_{R247A-R248A}$	pETM41-*fseA* with *fseA::R247A-R248A*	This study

Table S3. DNA primers used to prepare DNA probes for EMSAs

Primer name	Sequence 5′-3′	Description
rdfSp18_FL_R	GAACGAGGTCGTCGTGGGGA	Reverse primer for PCR amplification of the fluorescent P*rdfS* FseA-box DNA, 5′-IRDye700-tagged
rdfSp88_FL_F	CCGACGCTCGCCCGAAGG	Forward primer for PCR amplification of the fluorescent P*rdfS* FseA-box DNA, 5′-IRDye700-tagged
FseA_pMBPmt_R	AGGCTAGCAGGCCTACTAGTAAGCTTTTAGACGAGGAAATCCCGA	Reverse primer for PCR amplification of the non-specific DNA competitor
FseAmt_RR251AA_Fb	GCCGTGCCGTCGCGGCCGGTCACATGCTCATG	Forward primer for PCR amplification of the non-specific DNA competitor
rdfSp18_R	GAACGAGGTCGTCGTGGGGA	Reverse primer for PCR amplification of the specific DNA competitor
rdfSp91_F	CCGCCGACGCTCGCCCGAAGG	Forward primer for PCR amplification of the specific DNA competitor
pSDZ_prom_R	GCCTCTTCGCTATTACGCCAG	Reverse primer for PCR amplification of the fluorescent P*rdfS* IR DNA template
pSDZ_prom_F	TGTCGGTGAACGCTCTCCTG	Forward primer for PCR amplification of the fluorescent P*rdfS* IR DNA template

Table S4. Top 20 GREMLIN-predicted coevolving amino-acid pairs of FseA

i	j	i_id	j_id	r_sco	s_sco	prob
37	210	37_R	210_D	0.3086	2.914	1
212	254	212_S	254_H	0.2832	2.673	1
34	202	34_F	202_L	0.2772	2.617	1
218	250	218_H	250_V	0.265	2.502	1
205	252	205_V	252_R	0.2343	2.212	1
231	242	231_V	242_L	0.213	2.011	1
218	247	218_H	247_R	0.2124	2.006	1
44	72	44_D	72_Q	0.2043	1.929	1
26	144	26_T	144_Q	0.2033	1.92	1
33	36	33_E	36_R	0.2026	1.912	1
173	177	173_L	177_E	0.1985	1.874	0.999
13	211	13_W	211_G	0.1937	1.829	0.999
35	38	35_L	38_N	0.1893	1.787	0.999
205	256	205_V	256_L	0.1869	1.765	0.999
19	32	19_Y	32_W	0.1842	1.739	0.999
169	176	169_M	176_L	0.1831	1.729	0.999
207	211	207_Q	211_G	0.1806	1.705	0.998
13	19	13_W	19_Y	0.1803	1.702	0.998
36	41	36_R	41_F	0.1757	1.659	0.998
227	231	227_A	231_V	0.1752	1.654	0.998

i and j represent predicted coevolving residue pair positions, while i_id and j_id show the respective residues. Raw score is represented by r_sco, and the scaled score s_sco. Scaled score is the normalised raw score, calculated by dividing a pair's raw score by the average raw score. Prob denotes probability of the residues being in contact.

Table S5. NMR and refinement statistics for protein structures for 6H-QseM

**Distance and dihedral constraints**	
Total NOE	1177
Intra-residue	249
Inter-residue	928
Sequential (|*i* – *j*| = 1)	282
Medium-range (|*i* – *j*| < 4)	337
Long-range (|*i* – *j*| > 5)	309
Intermolecular	0
Hydrogen bonds	32
Total dihedral angle restraints	801
ϕ	225
ψ	225
**Structure statistics**	
Violations (mean and s.d.)	7
Distance constraints (Å)	4
Dihedral angle constraints (º)	3
Max. dihedral angle violation (º)	10.35
Max. distance constraint violation (Å)	0.29
**Deviations from idealized geometry**	
Bond lengths (Å)	0
Bond angles (º)	0
Impropers (º)	0
**Average pairwise r.m.s. deviation** (Å)**	
Heavy	0.88
Backbone	0.35

**Pairwise r.m.s. deviation was calculated among 20 refined structures.

REFERENCES

1. Studier,F.W. and Moffatt,B.A. (1986) Use of bacteriophage T7 RNA polymerase to direct selective high-level expression of cloned genes. *J. Mol. Biol.*, **189**, 113–130.

2. Robichon,C., Luo,J., Causey,T.B., Benner,J.S. and Samuelson,J.C. (2011) Engineering *Escherichia coli* BL21(DE3) derivative strains to minimize *E. coli* protein contamination after purification by immobilized metal affinity chromatography. *Appl. Environ. Microbiol.*, **77**, 4634–4646.

3. Simon,R., Priefer,U. and Pühler,A. (1983) A broad host range mobilization system for *in vivo* genetic engineering: Transposon mutagenesis in gram negative bacteria. *Nat. Biotechnol.*, **1**, 784–791.

4. Thoma,S. and Schobert,M. (2009) An improved *Escherichia coli* donor strain for diparental mating. *FEMS Microbiol. Lett.*, **294**, 127–132.

5. Sullivan,J.T. and Ronson,C.W. (1998) Evolution of rhizobia by acquisition of a 500-kb symbiosis island that integrates into a *phe*-tRNA gene. *Proc. Natl. Acad. Sci. U.S.A.*, **95**, 5145–5149.

6. Ramsay,J.P., Sullivan,J.T., Stuart,G.S., Lamont,I.L. and Ronson,C.W. (2006) Excision and transfer of the *Mesorhizobium loti* R7A symbiosis island requires an integrase IntS, a novel recombination directionality factor RdfS, and a putative relaxase RlxS. *Mol. Microbiol.*, **62**, 723–734.

7. Ramsay,J.P., Tester,L.G.L., Majora,A.S., Sullivan,J.T., Edgar,C.D., Kleffmann,T., Patterson-House,J.R., Hall,D.A., Tatec,W.P., Hynes,M.F., *et al*. (2015) Ribosomal frameshifting and dual-target antiactivation restrict quorum-sensing-activated transfer of a mobile genetic element. *Proc. Natl. Acad. Sci. U.S.A.*, **112**, 4104–4109.

8. Miller,W.G., Leveau,J.H.J. and Lindow,S.E. (2000) Improved *gfp* and *inaZ* broad-host-range promoter-probe vectors. *Mol. Plant-Microbe Interact.*, **13**, 1243–1250.

9. Ramsay,J.P., Bastholm,T.R., Verdonk,C.J., Tambalo,D.D., Sullivan,J.T., Harold,L.K., Panganiban,B.A., Colombi,E., Perry,B.J., Jowsey,W., *et al*. (2022) An epigenetic switch activates bacterial quorum sensing and horizontal transfer of an integrative and conjugative element. Nucleic Acids Res., **50**, 975–988.

10. Ramsay,J.P., Williamson,N.R., Spring,D.R., and Salmond,G.P. (2011) A quorum-sensing molecule acts as a morphogen controlling gas vesicle organelle biogenesis and adaptive flotation in an enterobacterium. *Proc. Natl. Acad. Sci. U.S.A*., **108**, 14932-14937.
